# Ocean warming and acidification synergistically increase coral mortality

**DOI:** 10.1038/srep40842

**Published:** 2017-01-19

**Authors:** F. Prada, E. Caroselli, S. Mengoli, L. Brizi, P. Fantazzini, B. Capaccioni, L. Pasquini, K. E. Fabricius, Z. Dubinsky, G. Falini, S. Goffredo

**Affiliations:** 1 Marine Science Group, Department of Biological, Geological and Environmental Sciences, University of Bologna, Via Selmi 3, I-40126 Bologna, Italy; 2 Department of Management, University of Bologna, Via Capo di Lucca 34, I-40126 Bologna, Italy; 3 Department of Physics and Astronomy, University of Bologna, Viale Berti Pichat 6/2, I-40127 Bologna, Italy; 4 Museo Storico e Centro Studi e Ricerche Enrico Fermi, Piazza del Viminale 1, I-00184 Roma, Italy; 5 Department of Biological, Geological and Environmental Sciences, University of Bologna, Piazza di Porta S. Donato 1, I-40127 Bologna, Italy; 6 Australian Institute of Marine Science, PMB 3, Townsville 4810, Queensland, Australia; 7 The Mina and Everard Goodman Faculty of Life Sciences, Bar–Ilan University, 52900 Ramat–Gan, Israel; 8 Department of Chemistry “G. Ciamician”, University of Bologna, Via Selmi 2, I-40126 Bologna, Italy

## Abstract

Organisms that accumulate calcium carbonate structures are particularly vulnerable to ocean warming (OW) and ocean acidification (OA), potentially reducing the socioeconomic benefits of ecosystems reliant on these taxa. Since rising atmospheric CO_2_ is responsible for global warming and increasing ocean acidity, to correctly predict how OW and OA will affect marine organisms, their possible interactive effects must be assessed. Here we investigate, in the field, the combined temperature (range: 16–26 °C) and acidification (range: pH_TS_ 8.1–7.4) effects on mortality and growth of Mediterranean coral species transplanted, in different seasonal periods, along a natural pH gradient generated by a CO_2_ vent. We show a synergistic adverse effect on mortality rates (up to 60%), for solitary and colonial, symbiotic and asymbiotic corals, suggesting that high seawater temperatures may have increased their metabolic rates which, in conjunction with decreasing pH, could have led to rapid deterioration of cellular processes and performance. The net calcification rate of the symbiotic species was not affected by decreasing pH, regardless of temperature, while in the two asymbiotic species it was negatively affected by increasing acidification and temperature, suggesting that symbiotic corals may be more tolerant to increasing warming and acidifying conditions compared to asymbiotic ones.

Under a business-as-usual scenario, climate change is expected to be associated to an increase of oceanic surface temperature by more than 3 °C and a decrease of global mean sea surface pH up to 0.32 units by the year 2100^1^. Ocean warming (OW) and acidification (OA) are two of the main stressors causing significant changes in marine environments, posing a major threat to species that generate and accumulate calcium carbonate structures, such as corals, potentially reducing the socioeconomic benefits of ecosystems reliant on calcifying organisms^2^,^3^,^4^.

The Mediterranean basin will likely be one of the most affected regions by climate change making it a miniature model of global patterns to occur in the world’s marine biota, and a natural focus of interest for research^5^. High temperatures have already caused several mass bleaching and mortality events in the Mediterranean^6^,^7^,^8^,^9^ and globally^10^. In order to correctly model and predict the effects of OW and OA it is important to investigate the possible combined effects of these two CO_2_-driven stressors^11^,^12^,^13^,^14^,^15^,^16^,^17^,^18^, which can affect organisms in different ways^19^,^20^: through additive effects (sum of the individual effects), synergistic effects (increased stress greater than the sum of the individual effect), and antagonistic effects (decreased stress).

Natural environmental gradients such as latitudinal temperature/solar radiation gradients and pH gradients found at CO_2_ vents have been used as “natural laboratories” as they more aptly represent conditions (nutrients, currents and irradiance) difficult or impossible to simulate *ex situ*^21^. Along an 850-km latitudinal gradient on the Western Italian coasts, increasing temperature negatively affects natural populations of the solitary zooxanthellate (i.e., symbiotic) Mediterranean coral *Balanophyllia europaea* (Risso, 1826), inducing a reduction of the net calcification rate, which results in a progressive decrease of skeletal bulk density and an increase in skeletal porosity, especially of larger sized pores. This determines a decrease of the resistance of the skeleton to mechanical stress. Furthermore, its population stability and abundance decrease with increasing temperature, as evidenced by a progressive lack of juveniles. On the other hand, the solitary non-zooxanthellate (i.e., asymbiotic) coral *Leptopsammia pruvoti* Lacaze-Duthiers, 1897 appears to be insensitive to the occurring temperature changes^22^,^23^,^24^,^25^,^26^,^27^,^28^. Gross calcification rate (CaCO_3_ production by the coral) of *B. europaea* transplanted at CO_2_ vents off Ischia, decreased with increasing acidity when the water was warmest^29^. Moreover, *B. europaea* naturally occurring at a CO_2_ vent off Panarea, under long-term exposure to acidic conditions, shows a significant decrease in population density and net calcification rate, accompanied by an increase in skeletal porosity, with increasing acidity^30^,^31^. This suggests that OA could likely exacerbate the mass benthic mortality events already recorded in the warming Mediterranean Sea, making it crucial to study the possible interactive effects between increasing sea temperature and acidity on coral mortality and growth rates.

In the present study, we investigated from July 2010 to April 2012 the effects of *in situ* exposure to different pH levels (range pH_TS_ 7.4–8.1) and seasonal temperatures (range 15.5–25.6 °C) on the mortality and net calcification rates of Mediterranean scleractinian corals transplanted near a volcanic CO_2_ vent off Panarea Island (Aeolian Islands, southern Italy) which generates a pH gradient at ambient temperature (see Supplementary Fig. S1 and Table S1). Transplant experiments are commonly used and have resulted in high quality studies^29^, because organisms are kept in a natural setting exposed to multiple environmental parameters which are difficult to simulate *ex situ.* Moreover, this approach allows exposing the organisms to more than one parameter at the time to study their possible combined effect. We transplanted the photosymbiont bearing solitary coral *B. europaea*, the solitary asymbiotic coral *L. pruvoti*, and the colonial asymbiotic coral *Astroides calyculari*s (Pallas, 1766) (see Supplementary Figs S2 and S3). Since coenosarc tissue of colonial corals was seen to break down with decreasing pH, leading to polyp dissociation^32^, for *A. calyculari*s we also measured coenosarc-polyp tissue mortality.

Predicting the combined effects of warming and acidification is not straightforward, as warming could either offset the effects of increased ocean acidity^33^ or exacerbate them through the addition of the single stress effects^34^. According to the self-extending symbiosis theory, symbiont-bearing organisms should be more tolerant to environmental change compared to organisms who do not host symbionts^35^. Moreover, colonial corals generally grow faster than solitary ones^36^, and fast-growing corals have been hypothesized to be more sensitive to acidification than slow-growing corals^37^. The above mentioned considerations led us to include in our experimental design solitary, colonial, zooxanthellate and non-zooxanthellate corals in order to investigate possible different responses to increased temperature and acidity.

## Results

### Seawater carbonate chemistry

Of the seawater parameters measured (pH, total alkalinity, temperature, and salinity), only pH differed significantly across sites (*P* < 0.001; see Supplementary Table S1 and Fig. S4). Even though fluctuations were observed, the average value decreased from 8.1 (min = 7.82; max = 8.45) at Site 1 to 7.4 (min = 6.71; max = 8.14) at Site 4. As shown in the supplementary information (see Supplementary Fig. S4), the pH gradient was maintained during the different transplant periods and did not show any seasonality. The pH changes were accompanied by significant shifts in carbonate-bicarbonate equilibria, with average aragonite saturation (Ω_arag_) decreasing by more than 60% from Site 1 (average = 3.6; min = 1.8; max = 6.3) to Site 4 (average = 1.4; min = 0.2; max = 3.1) (see Supplementary Fig. S1 and Table S1).

### Coral mortality and net calcification rates

**Figure 1:**
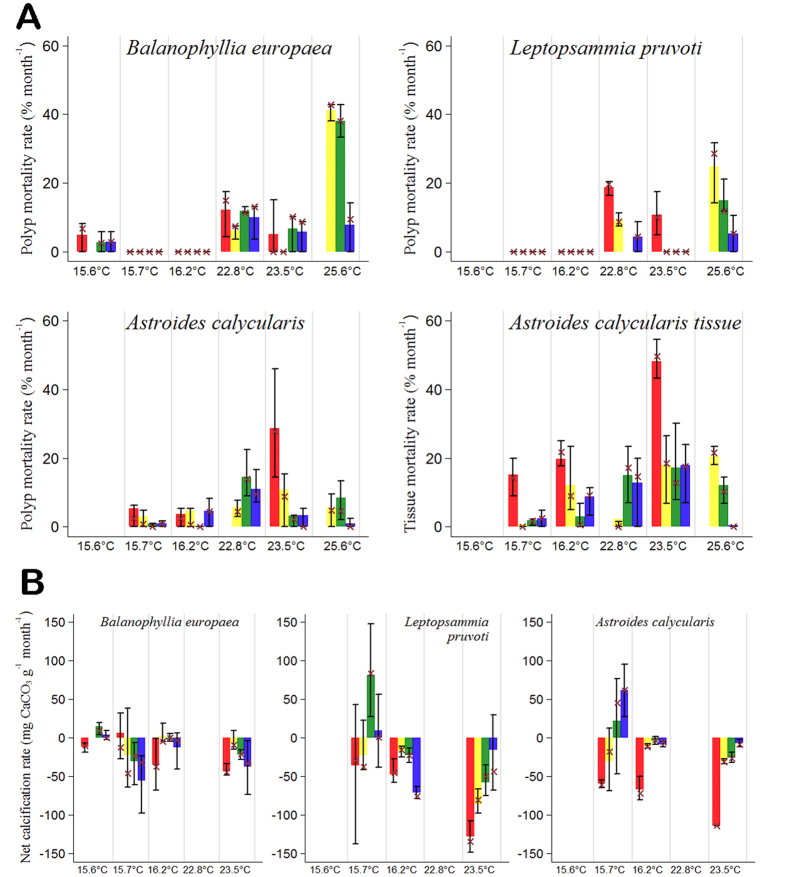
Plots of the values of mortality and net calcification rates for each average pH_TS_ value of each site and for each transplantation period for the three coral species. The acidity is indicated by the different colours: red (Site 4 - pH_TS_ 7.40), yellow (Site 3 - pH_TS_ 7.74), green (Site 2 - pH_TS_ 7.87), blue (Site 1 - pH_TS_ 8.07). Each transplantation period is denoted by the average seawater temperature. Average values = height of the histogram bars; median = cross, 10–90 percentiles intervals = error bars. Empty bins in the histograms indicate missing data due to storms that swept away the experiments. (**A**) Mortality rates (% month^−1^). Polyp mortality rate for *B. europaea*, *L. pruvoti*, and *A. calycularis* and coenosarc-polyp tissue mortality for the latter. For *B. europaea* and *L. pruvoti* at 15.7 °C and 16.2 °C and for the latter also at 23.5 °C the cross symbols indicate zero mortality. (**B**) Net calcification rates (mg CaCO_3_ g^−1^ month^−1^) for *B. europaea*, *L. pruvoti*, and *A. calycularis*.

The histograms for mortality and net calcification rates at each site and transplantation period for each species are reported in Fig. 1A and B, respectively. Empty bins in the histograms indicate missing data due to storms that swept away the experiments. To initially explore the data in testing the influence of seawater temperature (SWT), pH, and their interaction on mortality and net calcification rates, a standard two-way ANOVA (Table 1A) was applied to the data of Fig. 1. Table S2 shows the number of observations for each experimental period. To further investigate the interaction between pH and SWT, the experimental data were fitted to 3-dimensional functions where the independent variables are pH and SWT and the dependent variables are in one case mortality rate and in the other net calcification rate. Predicted parameters by the fitting functions for mortality and net calcification rates against pH and SWT for the three species are presented in Table 1B.

**Figure 2:**
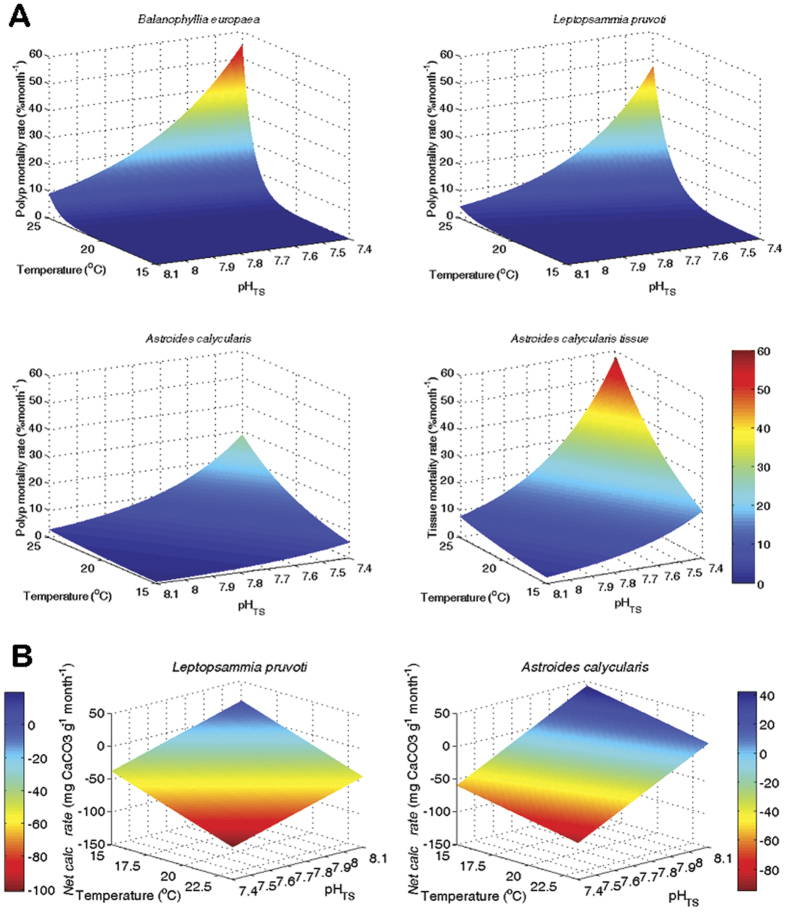
Plots of the three-dimensional functions obtained by regressions of mortality and net calcification rates for *B. europaea, L. pruvoti,* and *A. calycularis* against pH_TS_ and sea water temperature (°C), using the regression parameters in Table 1B. (**A**) 3-D plots of mortality rates (% month^−1^) (GLM log link functions). Polyp mortality rate for *B. europaea*, *L. pruvoti*, and *A. calycularis* and coenosarc-polyp tissue mortality for the latter. (**B**) 3-D plots of net calcification rates (mg CaCO_3_ g^−1^ month^−1^) (OLS linear functions). The plot for *B. europaea* is not reported because the fit in Table 1B showed no relationship with SWT and pH_TS_.

The histograms in Fig. 1A suggest that mortality rates increased with increasing SWT. Even though some mortality occurred at low temperatures, in some cases (i.e., *B. europaea* and *L. pruvoti* at average temperatures 15.7 and 16.2 °C) no mortalities were observed. Figure 1A also suggests that mortality rates increased with decreasing pH, and this effect was more pronounced at higher compared to lower temperatures. These observations are in agreement with the results of the two-way ANOVA test shown in Table 1A, confirming that polyp mortality rates in the three species and coenosarcs-polyp tissue mortality rate of *A. calycularis* increased significantly with increasing temperature (*P* < 0.001) and with decreasing pH (*P* < 0.001). Moreover, the significant interaction term indicated that both SWT and pH act synergistically (*P* < 0.001). Table 1B shows fitting parameters obtained by log link functions of mortality rates against pH and SWT for the three species. The goodness of fit to the model (χ^2^) resulted highly significant in all cases (*P* < 0.001; Table 1B). Polyp mortality for the three species and coenosarc-polyp tissue mortality had a negative relationship with pH (*P* < 0.001) and a positive relationship with SWT (*P* < 0.001). The 3-dimensional plots (Fig. 2A), obtained by plotting the predicted observations shown in Table 1B, show increased polyp mortality rates in all species and increased coenosarc-polyp tissue mortality rate in *A. calycularis* at low pH and high temperature. These plots visually show the single and combined effects of temperature and pH on mortality. The synergistic effect between pH and temperature is stronger than the effect of single variables because i.e., for a given pH range, the slope of the mortality rate became steeper with increasing temperature.

The plots in Fig. 1B suggest a difference in the behavior of the net calcification rate between the symbiotic *B. europaea* and the two asymbiotic species, *L. pruvoti* and *A. calycularis*. While in *B. europaea* the net calcification rate does not seem to show differences among pH treatments, regardless of temperature, in the asymbiotic species the net calcification rate seems to decrease with increasing acidity and temperature. The two-way ANOVA results shown in Table 1A confirmed these results showing no significant effects for *B. europaea*, while for *L. pruvoti* and *A. calycularis*, SWT and pH significantly affected net calcification rates separately (from *P* < 0.05 to *P* < 0.001; Table 1A). The interaction term was not significant indicating that SWT and pH act additively and not synergistically for net calcification rate (*P* > 0.05). Table 1B shows the predicted parameters of the net calcification rate to linear models for all the data. For *B. europaea* the goodness of fit to the model (F) was not significant (*P* > 0.05) while for *L. pruvoti* and *A. calycularis* it was highly significant (*P* < 0.001; Table 1B). For *B. europaea*, the net calcification rate was not affected by pH (*P* > 0.05) and had a negative relationship with SWT (*P* < 0.05). For *L. pruvoti* and *A. calycularis* the net calcification rate had a positive relationship with pH (from *P* < 0.05 to *P* < 0.001) and negative with temperature (*P* < 0.01). The 3-dimensional graphs (Fig. 2B), obtained by plotting the predicted observations shown in Table 1B, for net calcification rate in *L. pruvoti* and *A. calycularis,* show linear relationship upon increasing temperature and decreasing pH and visualize the additive (linear) and not synergistic effect for net calcification rates in our experimental conditions. The data for *B. europaea* was not plotted as the overall model resulted not significant.

SEM images (see Supplementary Fig. S5) of the skeletons of the three species, at low and high pH, in colder (average temperature 15.7 °C) and warmer (average temperature 22.0 °C) periods seem to show a smoothing of the surface under lowest pH, especially under high temperature. In all species, *septae* seem to be more brittle under high pH. In particular, the skeletal microstructures of *A. calycularis* seem particularly disordered under high temperature and low pH.

## Discussion

The distance of Panarea island from mainland (~60 Km) guarantees low interaction with nearshore waters. Thus, factors that might be relevant in unprotected areas (e.g., water quality, dysfunctions in trophic interactions linked to overfishing)^38^, were excluded in the present study. There is also no evidence that food availability or irradiance differed among Sites, as Site 1 is only 34 m from Site 4 and at approximately the same depth. The strong currents that generate the pH gradient ensure a regular rapid water exchange. Possible contaminants (e.g., metals) can be excluded because emissions are exclusively gaseous at ambient temperature, and dissolved H_2_S in the four sites was below detection limit (see Goffredo *et al*.^30^).

Increased temperature and acidity had a strong synergistic effect on polyp mortality rate in all three species and on coenosarcs-polyp tissue mortality rate of the colonial species. The mortality rates were higher at lower pH when temperatures were higher. The observed detrimental effect of temperature in warm months could lie on reduced food (energy) availability in summer. However, seasonal plankton patterns studied over 14 years at a coastal station in the Gulf of Naples (southern Tyrrhenian Sea) show that mesozooplankton reached the highest values of biomass and abundance in mid spring (April-May) and summer (July-September), and the lowest values in winter (December and January)^39^. Further studies are needed to elucidate feeding habits of these species (currently unknown) and whether seasonal plankton community fluctuations in our experimental setting may influence energy supply in these species throughout the year. Another hypothesis could depend on the effect of different current regimes among seasons on the thickness of the Diffusion Boundary Layer (DBL: the boundary between the organism and the surrounding environment)^40^ of the transplanted corals. Water flowing across corals enhances gas and metabolite exchange crucial for their nurture, growth and reproduction^41^,^42^. Thus, slow water motion during summer periods could thicken the DBL leading to hypercapnia and in turn to metabolic stress (e.g., symbionts or cell processes producing large amounts of organic waste, higher cell respiration)^43^. However, the stability of the main current (from North/West to South/East) that creates the Panarea pH gradient was confirmed during the different expeditions performed for this experiment and also by other studies conducted in the same area^44^,^45^. Thus, to the best of our knowledge, there isn’t a time of the year in which, in our experimental setting, water motion is still, leading to a significant thickening of the DBL. However, the latter and also other aspects (e.g., possible heat shock responses, relevant skeletal and/or cellular proteins out of their thermodynamic optimum/range which could lead to incorrect protein folding, cellular waste and build-up of toxic substances) deserve further investigation to unravel the observed mortality increases in warmer months.

Also coenosarc-polyp tissue mortality in *A. calycularis* was highly sensitive to increasing temperature and acidity (Fig. 1A). In a previous study by Kvitt *et al*.^46^, incubation of two colonial coral species (*Pocillopora damicornis* and *Oculina patagonica*) at reduced pH induced coenosarc breakdown and loss of coloniality^46^, suggesting a mechanistic model in which colonial corals rid themselves of energetically costly processes (e.g., calcification, tissue maintenance) through tissue breakdown. Long-term exposure to elevated temperatures causes physiological stress to benthic species, such as enhanced respiration^47^, higher susceptibility to pathogens^48^, bleaching^49^, reduced calcification^50^ and tissue necrosis^47^,^50^,^51^. Tissue breakdown and mortality in Mediterranean corals have already been linked to elevated summer temperatures^8^,^50^,^52^,^53^. In particular, mass mortality events have already hit *A. calycularis* populations in the Tyrrhenian Sea exposed to unusually high temperatures (up to 28–29 °C) in summer of 2009 at Ischia^54^.

Our results confirm that *A. calycularis* seems particularly sensitive to high summer temperatures, displaying an increase in tissue mortality to such an extent that this in turn probably made the corals more susceptible to the detrimental effects of ocean acidification on net calcification. In fact, *A. calycularis* seems to show the most severe effects on net calcification (Table 1), as confirmed also by the highly disordered skeletal microstructures under high temperature and low pH (see Supplementary Fig. S5).

Adverse effects on net calcification rates were observed only in the asymbiotic species. *In-vitro* CaCO_3_ deposition experiments showed that *B. europaea* intra-skeletal organic matrix favours the precipitation of aragonite even in the absence of Mg ions, while in *L. pruvoti* and *A. calycularis* this does not happen^55^. This observation indicates that in *B. europaea* a higher involvement of intra-skeletal organic matrix macromolecules over the mineralization process is present, which could perhaps be related to the observed reduced *B. europaea* sensitivity to temperature and acidification conditions compared to *L. pruvoti* and *A. calycularis*. Moreover, net calcification is the result of CaCO_3_ production (gross calcification) and dissolution. It has been previously observed^29^ that in *B. europaea* gross calcification in an acidic environment increases at lower temperatures, when dissolution is thermodynamically favored. Our results are consistent with a balance of the two effects in *B. europaea,* who seems to modulate gross calcification keeping net calcification unaltered, regardless of pH or temperature. A possible explanation could be that the symbiosis with zooxanthellae could make *B. europaea* more tolerant than asymbiotic species in a hypercapnic (elevated CO_2_) environment. Calcification is tightly linked to photosynthesis by the symbiotic zooxanthellae^56^,^57^,^58^,^59^,^60^. Additionally, photosynthetic removal of CO_2_ by the zooxanthellae might help mitigate the effects of acidification^61^. A further explanation could be that *B. europaea* may be able to better up-regulated the pH at the site of calcification relative to seawater compared to the other two species likely fostering calcification. This species-dependent pH-buffering capacity is not ubiquitous among calcifying organisms so those lacking this ability will more likely undergo declines in calcification under ocean acidification scenarios^33^. Elevated CO_2_ also affects metabolic rates by shifting the steady state acid-base equilibria^62^, reducing relevant transmembrane ion exchange rates^63^ and limiting protein synthesis rates with long-term detrimental effects on growth and reproduction^64^. The extracellular acid–base status responds in a species-specific way so we cannot exclude that *B. europaea* may have a greater acid-base stability compared to *L. pruvoti* and *A. calycularis.* An additional explanation could depend on the fact that, in light, calcification is higher in zooxanthellate than in non-zooxanthellate corals^65^,^66^,^67^. Several studies have demonstrated that the pH of the diffusive boundary layer (DBL) surrounding corals could be influenced by respiration and photosynthesis^68^. Higher day-time calcification rates in the zooxanthellate coral could compensate the negative effect on calcification of night time hypercapnic conditions of the DBL induced by respiration^69^, while the non-zooxanthellate species may lose CaCO_3_ also during daytime. In the latter species the DBL may be CaCO_3_ undersaturated almost all the time when circumfluent seawater near the CO_2_ vent is already close to undersaturation. Further studies at cellular and molecular levels on internal-external exchange processes/ion sym- or antiporters at the cell wall (e.g., Ca^2+^), or calcifying tissue layer (i.e., ion exchange with the seawater/the diffusive boundary layer), pH homeostasis and gene expression are needed to elucidate the mechanisms underlying the observed responses of calcification to increased acidity among species. In conclusion, we have reported two main findings. (i) In all three species, high temperature exacerbates the negative effect of decreasing pH on mortality rates, with the highest mortalities during the warmest periods. It is likely that high seawater temperatures increased their metabolic rates (e.g., respiration rates) up to a point that, in conjunction with decreasing pH, could have led to rapid deterioration of cellular processes and performance. This study confirms previous observations on coral growth indicating that calcifying organisms may be more vulnerable to the effects of OA when the water is warmest^29^,^70^, contributing to a growing body of evidence that shows how combined warming and acidifying conditions predicted in the coming decades will likely be detrimental to important components of shallow water ecosystems. (ii) The net calcification rate of the symbiotic *B. europaea* was not affected by decreasing pH, regardless of temperature, while for the two asymbiotic species *L. pruvoti* and *A. calycularis* it was negatively affected by decreasing pH and increasing temperature, suggesting that *B. europaea* may be more tolerant to increasing acidity compared to the other two species. Investigations at Panarea^30^,^31^ and Ischia CO_2_ vents^29^,^71^ have shown that at pH_TS_ 7.8, an ecological tipping point occurs as corals and mollusks disappear below this threshold. Our *in situ* transplant experiment is in agreement with this tipping point as in most cases we reported the highest mortality and the lowest calcification rates at mean pH_TS_ ≤ 7.7. The fact that no relationship between net calcification and decreasing pH was observed, regardless of temperature, in the transplanted *B. europaea* corals suggests that under short-term (a few months) exposure this species may be able to somehow better cope, compared to the two asymbiotic species, under hypercapnic conditions. However, this capability is disrupted when the species is subjected to long-term (years) acidified conditions, which result in decreased net calcification and subsequent inability to survive below pH_TS_ 7.8^30^,^31^. Further seasonal experimental studies are needed on the cellular processes (e.g., cell metabolism, molecular calcification) in different coral species under OW and OA to better understand what makes some species “losers or winners” to rising seawater temperature and decreasing pH.

## Materials and Methods

### Study site and observation periods

The experimental site, which has been previously described by Goffredo *et al*.^30^, is located off the Island of Panarea (Aeolian Islands, southern Italy). A crater (20 × 14 m) at 10 m depth generates a stable sustained column of bubbles (98–99% CO_2_, 0.2–0.3% N_2_, 0.01–0.02% O_2_, 0.003–0.005% Ar, 0.001–0.002% CH_4_, 0.3–0.6%), at ambient temperature, creating a natural pH gradient extending for ~34 m along the direction of the main current (from North/West to South/East at speeds of 0.2–0.6 m sec^−1^)^44^. Four experimental sites were selected along the 34 m gradient (see Supplementary Fig. S1): Site 1 (mean pH_TS_ 8.1), Site 2 (mean pH_TS_ 7.9), Site 3 (mean pH_TS_ 7.7), and Site 4 (mean pH_TS_ 7.4). Data were collected in the following experimental periods (average temperature (95% CI): December 2011–April 2012 (15.6 (15.5–15.6) °C), November 2010–March 2011 (15.7 (15.5–15.7) °C), March–June 2011 (16.2 (16.0–16.3) °C), July–December 2011 (22.8 (22.6–23.0) °C), June–July 2011 (23.5 (23.4–23.7) °C), July–September 2010 (25.6 (25.5–25.7) °C).

### Carbonate chemistry

Temperature, salinity, pH (NBS scale) and total alkalinity were measured as previously described in ref. 30. Measured pH_NBS_ were converted to the total scale using CO2SYS software^72^. Mean pH_TS_ (back-transformed hydrogen ion concentrations) was calculated for each site. The pH_TS_, total alkalinity, salinity and temperature were used to calculate carbonate chemistry parameters using the software CO2SYS with referenced dissociation constants^73^,^74^,^75^. Temperature sensors (Thermochron iButton, DS1921G, Maxim Integrated Products, USA) attached at each site recorded depth temperature (°C) every three hours from June 2011 to May 2013. Sea surface temperatures (°C) at each Site were recorded hourly by mareographic stations close to Panarea Island using SM3810 (Society for Environmental and Industrial monitoring; SIAP + MICROS) from the National Mareographic Network of the Institute for the Environmental Protection and Research (ISPRA, available to http://www.mareografico.it). Linear regression analysis of depth temperature and sea surface temperature was used to estimate depth temperatures during transplantation periods (see Supplementary Table S3).

### Field transplantation and biotic measurements

During expeditions, similarly sized *B. europaea*, *L. pruvoti* and *A. calycularis* specimens collected ~2 km from the vent area were transplanted at the four sites (see Supplementary Fig. S1). The same number of corals were randomly assigned to each of the four sites. *Astroides calycularis* colonies were fixed with cable ties onto plastic grids; *B. europaea* and *L. pruvoti* polyps were glued with a bicomponent epoxy coral glue (Milliput, Wales, UK) onto ceramic tiles. *Leptopsammia pruvoti* polyps were placed upside-down under plastic cages to mimic their natural orientation in overhangs and caves. A total of ~950 solitary polyps and ~350 colonies with approximately 50 polyps per colony were considered in the present study. Samples were transplanted to the four sites for up to six seasonal periods (from July 2010 to April 2012), characterized by different average seawater temperatures in the range 15.5–25.6 °C (see Supplementary Table S1). After each transplant period the samples were collected, analyzed and stored, and new corals were transplanted for the following seasonal period. The duration of these periods ranged from 2 to 5 months due to logistic reasons. However, this does not affect the results, because the highest mortality was observed in the warmest periods, which were also the shortest.

Mortality rates (% month^−1^: polyp mortality rate for the three species, and coenosarc-polyp tissue mortality rate for the colonial *A. calycularis*) were assessed using digital photographs taken with a Canon G11 camera with underwater housing. Polyp mortality was determined by visually counting live polyps. A polyp was considered dead when live tissue was no longer visible. Coenosarc-polyp tissue mortality was determined by image analysis^76^ (see Supplementary Fig. S3). Coral net calcification rate (mg CaCO_3_ g^−1^ month^−1^), the result of CaCO_3_ production by the coral and dissolution by the environment, was measured using the buoyant weight technique^77^. Net calcification rate was the change in dry weight (calculated from the equation used in ref. 68) before and after each experimental period, normalized to initial weight and expressed as monthly variation (mg g^−1^ month^−1^).

### Low magnification SEM images

Cleaned coral skeletons from each species (experimental periods November 2010-March 2011 and May-June 2012), were mounted (uncoated) to conductive carbon tape and examined with a Tescan Vega3 GMU variable pressure scanning electron microscope. Two corals per site, per species, were analyzed.

### Statistical analysis

Environmental conditions between sites were compared using the non-parametric Kruskal-Wallis test (χ^2^). To initially run an exploratory analysis of the interaction between sea water temperature (SWT) and pH, we tested the influence of SWT, pH, and their interaction on the dependent variables (mortality rate and net calcification rate) with a two-way analysis of variance (ANOVA). The statistical significance was assessed by F-test. The presence of a significant interaction indicates that the effect of one predictor variable on the response variable is different at different values of the other predictor variable. It is tested by adding a term to the model in which the two predictor variables are multiplied (SWTxpH). Regression functions were used to further investigate the effects on mortality and net calcification rates of the predictor variables SWT and pH. For mortality data, to avoid possible misleading inferences due to the fact that classic distribution assumptions are violated (e.g., only positive values), we used a Generalized Linear Model (GLM). On the other hand, for net calcification these assumptions are met, thus we used a simpler model, the Ordinary Least Squares (OLS). For GLM the regression parameters are estimated via maximum likelihood which requires z-test to infer about the statistical significance of the parameters. For OLS the significance of regression parameters is estimated using a t-test. Mortality rates were estimated using a GLM log link function to constrain estimated values to be non-negative, and their variance was taken to be proportional to the mean. Statistical significance of the model was inferred using a χ^2^-test. OLS linear functions robust to outliers were used to estimate net calcification rates. The statistical significance of the model was assessed using an F-test. Statistical analyses were performed using STATA 12.0 (StataCorp LP).

## Additional Information

**How to cite this article**: Prada, F. *et al*. Ocean warming and acidification synergistically increase coral mortality. *Sci. Rep.* **7**, 40842; doi: 10.1038/srep40842 (2017).

**Publisher's note:** Springer Nature remains neutral with regard to jurisdictional claims in published maps and institutional affiliations.

## Supplementary Material

Supplementary Information

## Figures and Tables

**Table 1:** Statistical analyses of mortality and net calcification rates for corals transplanted in different seasonal periods and acidity sites.

	*B. europaea*	*L. pruvoti*	*A. calycularis*	*A. calycularis*	*B. europaea*	*L. pruvoti*	*A. calycularis*
	Polyp mortality	Polyp mortality	Polyp mortality	Tissue mortality	Net calcification	Net calcification	Net calcification
**(A)**							
SWT	73.94***	43.80***	16.14***	33.42***	3.19	8.95***	3.74*
pH	6.63***	12.84***	14.26***	41.27***	1.76	3.53*	8.23***
SWTxpH	9.36***	6.13***	7.03***	8.32***	1.46	2.13	1.27
N	66	54	214	209	83	64	47
R^2^	0.922	0.879	0.421	0.617	0.289	0.421	0.568
F	24.82***	15.32***	8.37***	18.07***	1.97	3.44**	4.17***
	GLM				OLS		
pH	−2.57***	−3.38***	−3.25***	−2.86***	−7.01	82.57*	145.67***
	(−4.50)	(−5.40)	(−7.23)	(−10.81)	(−0.36)	(2.19)	(7.49)
SWT	1.02***	0.70***	0.16***	0.12***	−2.11*	−7.36**	−4.04**
	(6.00)	(5.40)	(5.90)	(7.70)	(−2.03)	(−3.21)	(−3.23)
Const.	−2.39	11.37***	23.41***	22.22***	77.04	−539.19	1,077.44***
	(−0.76)	(4.00)	(7.57)	(11.80)	(0.50)	(−1.79)	(−7.91)
N	66	54	214	209	83	64	47
χ^2^	35.96***	32.01***	57.11***	126.6***			
R^2^					0.036	0.186	0.442
F					2.06	9.21***	29.76***

(**A**) Two-way analysis of variance (ANOVA) for the predictor variables sea water temperature (SWT, °C), pH (total scale), and for their interaction (SWT × pH). The predicted variables are polyp mortality rate (% month^−1^) of *B. europaea*, *L. pruvoti* and *A. calycularis*, tissue mortality rate of *A. calycularis*, and net calcification rate (mg CaCO_3_ g^−1^ month^−1^) of the three species. The statistical significance was assessed by F-test. (**B**) Regression analysis for mortality rate (% month^−1^) (through GLM log link functions) and net calcification rate (mg CaCO_3_ g^−1^ month^−1^) (through OLS linear functions) against pH (total scale) and SWT (°C) for *B. europaea, L. pruvoti* and *A. calyculari*s. Levels of significance: ***p < 0.001, **p < 0.01, *p < 0.05. χ^2^ and F were used to test the goodness of fit for GLM and OLS, respectively. N = for polyp mortality, number of tiles in *B. europaea* and *L. pruvoti,* and number of colonies in *A. calyculari*s; for net calcification, number of polyps in *B. europaea* and *L. pruvoti,* and number of colonies in *A. calyculari*s.
