# Supplementary material for: Ocean warming and acidification synergistically increase coral mortality
Source: Sci Rep. 2017 Jan 19;7:40842. doi: 10.1038/srep40842 (PMC5244398; doi:10.1038/srep40842)
Supplement: Supplementary Information [file srep40842-s1.pdf]

SUPPLEMENTARY INFORMATION

Ocean warming and acidification synergistically increase coral mortality

F. Prada, E. Caroselli, S. Mengoli, L. Brizi, P. Fantazzini, B. Capaccioni, L. Pasquini, K. E. Fabricius, Z. Dubinsky, G. Falini, S. Goffredo

SUPPLEMENTARY FIGURES

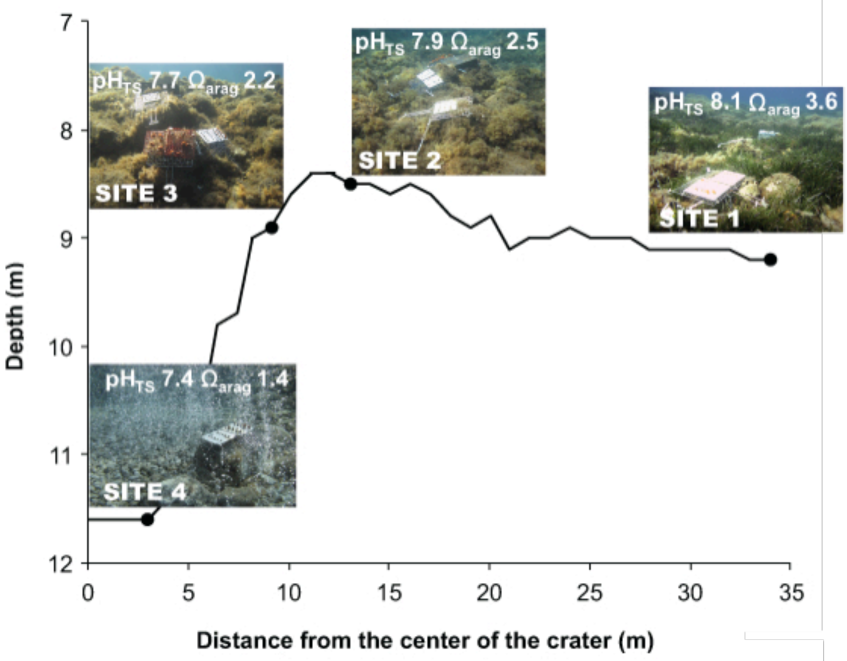

Fig. S1. Bathymetric profile of the four sites. Sites are shown in the pictures, with the associated mean pH_{TS} and Ω_{arag}.

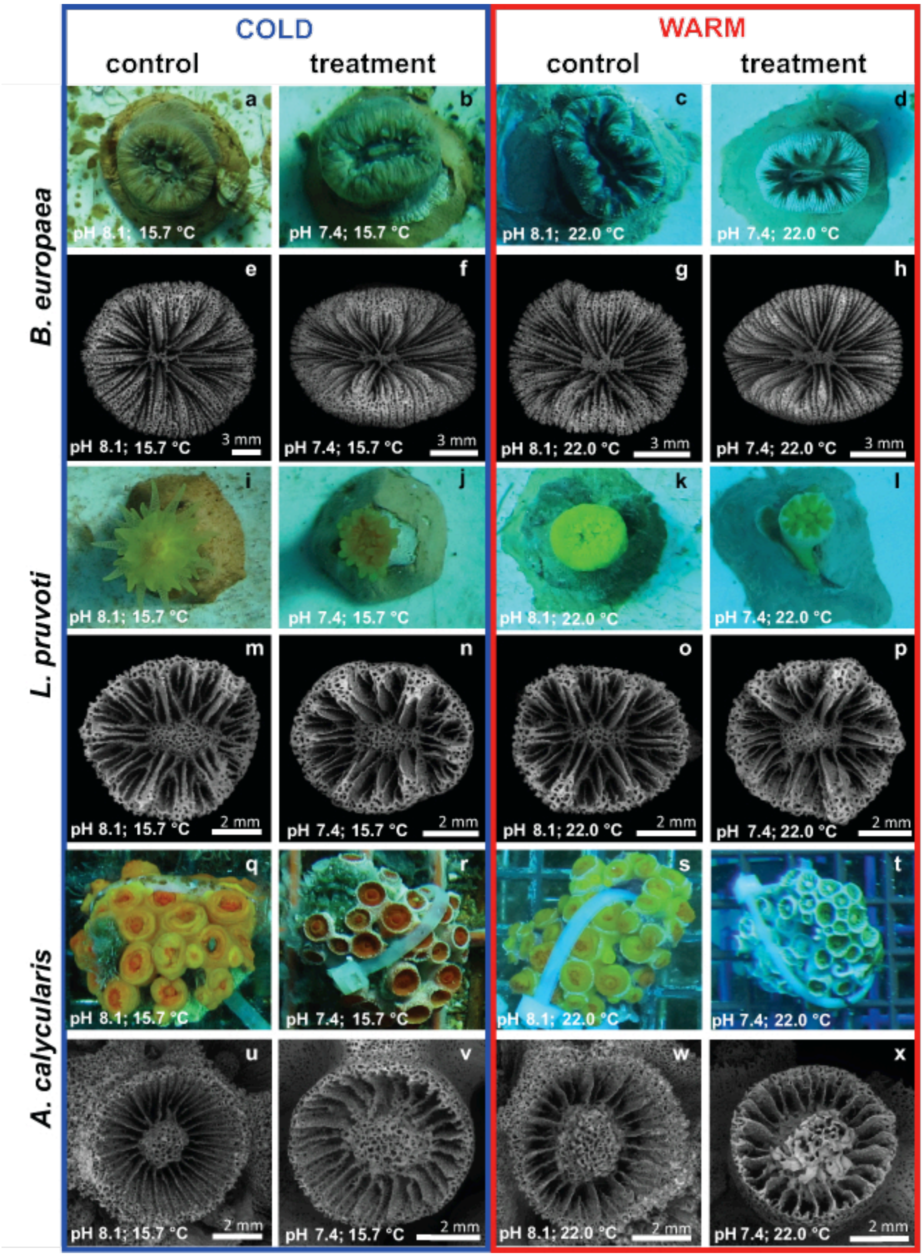

Fig. S2. Images of the three coral species under different pH and temperature conditions. **a-d**, **i-l**, **q-t**: Pictures of live *B. europaea*, *L. pruvoti,* and *A. calycularis* specimens. **e-h**, **m-p**, **u-x**: Low magnification SEM images of coral skeletons (image courtesy of J.C. Weaver at Harvard University). Images are reported for corals exposed to mean pH 8.1 and pH 7.4 in COLD (November 2010-March 2011; mean temperature: 15.7°C) and WARM (May-June 2012; mean temperature: 22.0°C) periods.

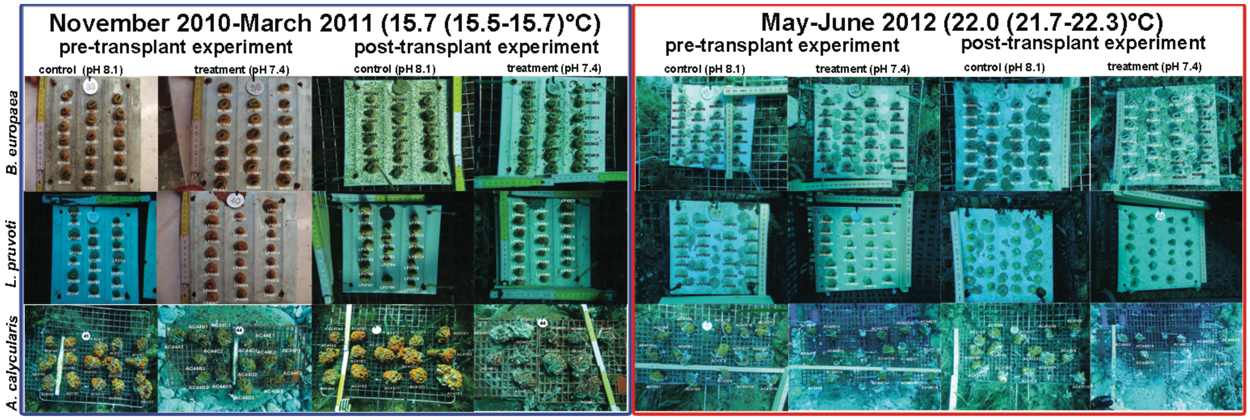

Fig. S3. Pre and post experimental images for *B. europaea*, *L. pruvoti* and *A. calycularis* under different pH and temperature conditions. A few examples are given of specimens at the beginning and at the end of transplant experiments in two representative transplant periods (COLD period in blue: November 2010-March 2011; WARM period in red: May-June 2012) and two sites (control: pH_{TS} 8.1; treatment: pH_{TS} 7.4).

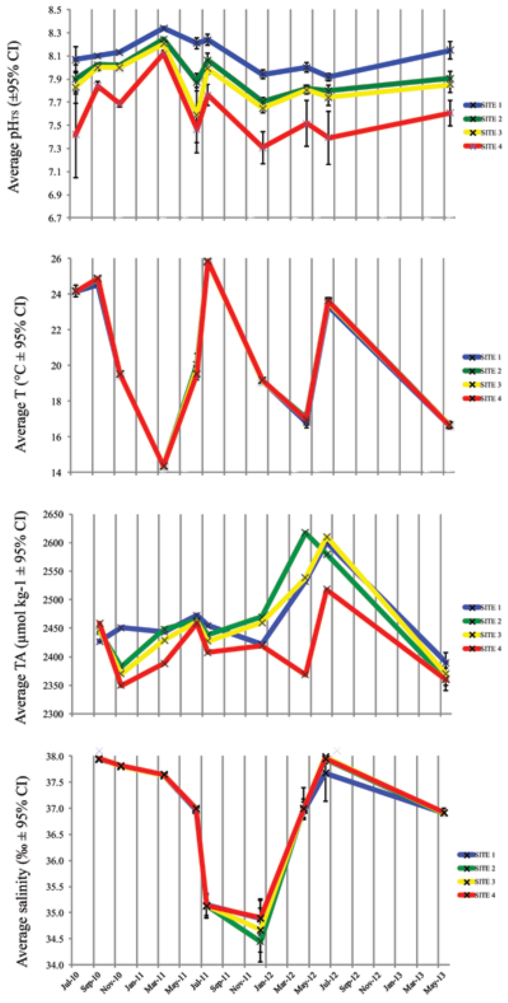

Fig. S4. Plots of average pH_{TS}, temperature, total alkalinity and salinity as measured at the times of data collection at the experimental sites. Average values and 95% confidence intervals were calculated from the related data reported in Table S1.

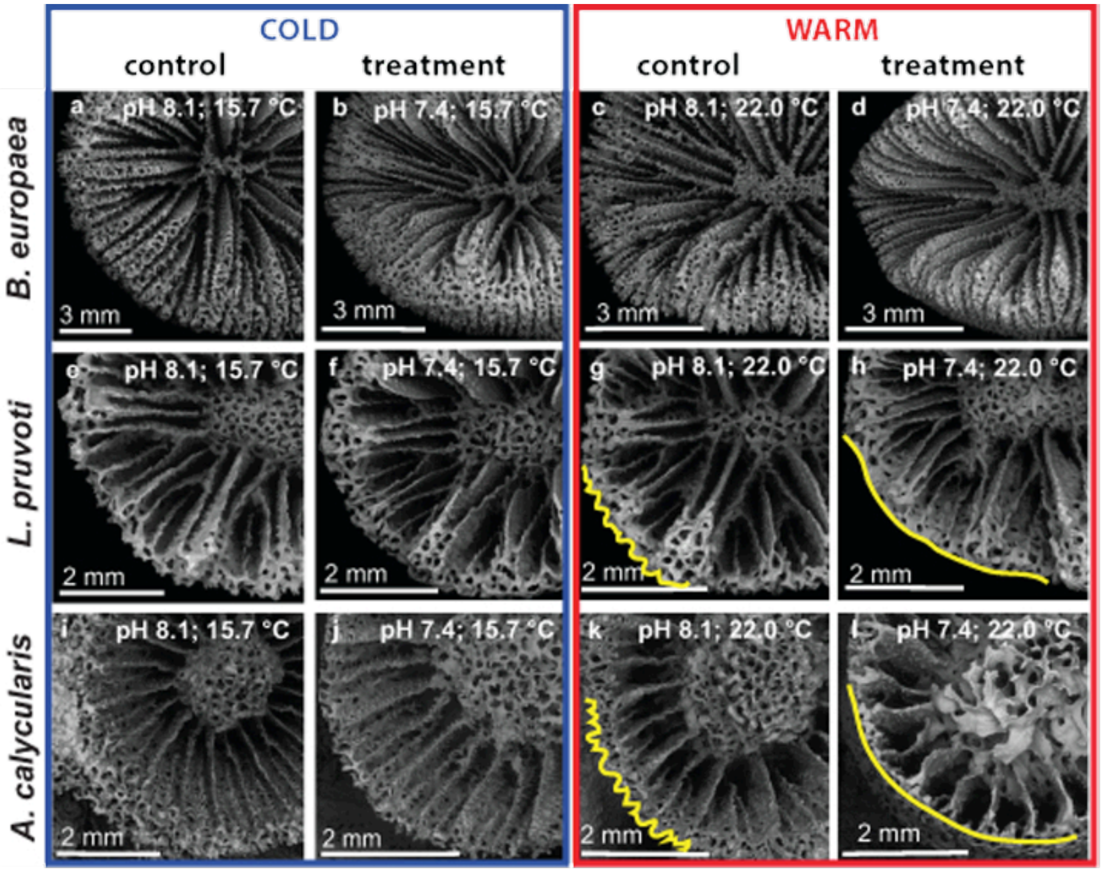

Fig. S5. Low magnification SEM images of skeletons. SEM images of skeletons of *Balanophyllia europaea, Leptopsammia pruvoti* and *Astroides calycularis* specimens under different pH and temperature conditions (image courtesy of J.C. Weaver at Harvard University). **a-d**, Images of *B. europaea*, **e-h**, *L. pruvoti*, and **i-l,** *A. calycularis* skeletons. **g, k,** Yellow contour lines highlight rough and **h, l,** smooth surfaces at mean pH 8.1 and pH 7.4, respectively, for *L. pruvoti* and *A. calycularis*. Images are reported for corals in COLD (November 2010-March 2011; mean temperature: 15.7°C) and WARM (May-June 2012; mean temperature: 22.0°C) periods.

SUPPLEMENTARY TABLES

Measured Parameters

Treatment	pH range (total scale)	T (°C)	TA (μmol kg^{-1})	S (‰)
Site 1	8.07 (7.82-8.45)	20.5 (14.3-26.0)	2438 (2368-2600)	37 (33-38)
Site 2	7.87 (7.54-8.25)	20.7 (14.4-26.0)	2429 (2334-2618)	37 (33-38)
Site 3	7.74 (7.05-8.21)	20.6 (14.4-26.0)	2426 (2343-2610)	37 (34-38)
Site 4	7.40 (6.71-8.14)	20.6 (14.4-26.0)	2395 (2329-2518)	37 (34-38)
**Statistical significance**	***	NS	NS	NS

Calculated Parameters

Treatment	*pCO_2 (μatm)	*HCO_3^- (μmol kg^{-1})	*CO_3^{2-} (μmol kg^{-1})	*DIC (μmol kg^{-1})	*Ω_{arag}
Site 1	391 (127-780)	1869 (1466-2144)	232 (120-398)	2114 (1867-2291)	3.6 (1.8-6.3)
Site 2	672 (234-1561)	2030 (1664-2264)	163 (68-314)	2214 (1984-2383)	2.5 (1.1-5.0)
Site 3	907 (262-5100)	2073 (1835-2365)	144 (25-243)	2246 (2089-2552)	2.2 (0.4-3.9)
Site 4	1944 (306-7231)	2159 (1826-2355)	96 (16-233)	2317 (2070-2613)	1.4 (0.2-3.1)
**Statistical significance**	***	***	***	***	***

Table S1. Seawater carbonate chemistry. Measurements are shown for each study Site off the Island of Panarea. Site 1 is the reference and sites 2-4 are the elevated pCO_2 Sites. Temperature (T; n = 112-115 per site), pH (n = 103-110 per site) and salinity (S; n = 107-110 per site) were measured in July 2010, September 2010, November 2010, March 2011, June 2011, July-August 2011, November-December 2011, April-May 2012, June 2012 and May 2013. Total alkalinity (TA; n = 14 per site) was measured in September 2010, November 2010, March 2011, June 2011, July-August 2011, November-December 2011, April-May 2012, June 2012 and May 2013. pCO_2 = carbon dioxide partial pressure; HCO_3^- = bicarbonate; CO_3^{2-} = carbonate; DIC = dissolved inorganic carbon; Ω_{arag} = aragonite saturation; NS = not significant; ***p<0.001, Kruskal-Wallis test. In brackets the min and max values.

	MORTALITY RATE					
	**Dec 2011-Apr 2012**	**Nov 2010-Mar 2011**	**Mar-Jun 2011**	**June-July 2011**	**July-Dec 2011**	**July-Sep 2010**
***B. europaea* polyp**	9	12	12	12	12	9
***L. pruvoti* polyp**		12	12	9	12	9
***A. calycularis* polyp**		45	46	44	35	44
***A. calycularis* tissue**		45	46	44	35	39

	NET CALCIFICATION RATE			
	**Dec 2011-Apr 2012**	**Nov 2010-Mar 2011**	**Mar-Jun 2011**	**July-Dec 2011**
B. europaea	18	24	20	21
L. pruvoti		21	21	22
A. calycularis		20	15	12

Table S2. Number of observations for each species and experimental period.

For polyp mortality, number of tiles in *B. europaea* and *L. pruvoti,* and number of colonies in *A. calycular*is; for net calcification, number of polyps in *B. europaea* and *L. pruvoti,* and number of colonies in *A. calycular*is.

**December 2011-April 2012**		**November 2010-March 2011**		**March-June 2011**		**July-December 2011**		**June-July 2011**		**July-September 2010**	
DATE	DEPTH T (°C)	DATE	DEPTH T (°C)	DATE	DEPTH T (°C)	DATE	DEPTH T (°C)	DATE	DEPTH T (°C)	DATE	DEPTH T (°C)
20111201	18.1	20101115	18.8	20110303	14.3	20110731	24.6	20110606	21.5	20100714	26.4
20111201	18.1	20101115	18.7	20110303	14.3	20110731	24.5	20110606	21.3	20100714	24.8
20111201	18.1	20101115	18.6	20110303	14.1	20110731	24.4	20110606	21.3	20100714	24.5
20111201	18.1	20101115	18.8	20110303	14.2	20110731	24.5	20110606	21.4	20100714	24.5
20111201	18.4	20101115	19.1	20110303	14.4	20110731	25.1	20110606	21.7	20100714	25.7
20111201	18.4	20101115	19.1	20110303	14.6	20110731	25.2	20110606	22.1	20100714	27.3
20111201	18.3	20101115	19.0	20110303	14.5	20110731	24.8	20110606	21.9	20100714	27.7
20111201	18.2	20101115	18.9	20110303	14.4	20110731	24.6	20110606	21.7	20100714	27.5
20111202	18.2	20101116	18.8	20110304	14.3	20110801	24.4	20110607	21.4	20100715	26.6
20111202	18.1	20101116	18.7	20110304	14.3	20110801	24.3	20110607	21.2	20100715	25.6
20111202	18.0	20101116	18.8	20110304	14.0	20110801	24.3	20110607	21.0	20100715	25.3
20111202	18.1	20101116	18.8	20110304	14.0	20110801	24.5	20110607	21.5	20100715	25.3
20111202	18.3	20101116	18.8	20110304	14.2	20110801	25.0	20110607	22.0	20100715	25.2
20111202	18.4	20101116	18.8	20110304	14.4	20110801	25.2	20110607	22.4	20100715	26.9
20111202	18.1	20101116	18.7	20110304	14.3	20110801	24.7	20110607	22.2	20100715	27.8
20111202	18.0	20101116	18.7	20110304	14.2	20110801	24.5	20110607	21.3	20100715	27.7
20111203	17.9	20101117	18.6	20110305	14.1	20110802	24.4	20110608	21.0	20100716	26.8
20111203	17.9	20101117	18.7	20110305	14.1	20110802	24.3	20110608	20.8	20100716	25.5
20111203	17.8	20101117	18.7	20110305	14.1	20110802	24.3	20110608	21.3	20100716	26.7
20111203	17.8	20101117	18.7	20110305	14.1	20110802	24.6	20110608	21.4	20100716	25.3
20111203	18.1	20101117	18.6	20110305	14.4	20110802	25.2	20110608	21.9	20100716	26.0
20111203	18.1	20101117	18.6	20110305	14.5	20110802	25.5	20110608	21.3	20100716	27.0
20111203	18.1	20101117	18.5	20110305	14.3	20110802	24.9	20110608	20.8	20100716	27.1
20111203	18.0	20101117	18.5	20110305	14.1	20110802	24.7	20110608	21.4	20100716	27.7
20111204	18.0	20101118	18.5	20110306	14.1	20110803	24.6	20110609	21.3	20100717	27.7
20111204	18.0	20101118	18.5	20110306	14.2	20110803	24.5	20110609	20.8	20100717	27.5
20111204	18.1	20101118	18.5	20110306	14.1	20110803	24.4	20110609	19.9	20100717	26.5
20111204	18.1	20101118	18.4	20110306	14.2	20110803	24.7	20110609	19.7	20100717	26.2
20111204	18.3	20101118	18.5	20110306	14.2	20110803	25.4	20110609	20.7	20100717	26.8
20111204	18.3	20101118	18.4	20110306	14.4	20110803	25.6	20110609	20.0	20100717	27.0
20111204	18.2	20101118	18.4	20110306	14.2	20110803	25.0	20110609	19.2	20100717	27.7
20111204	18.2	20101118	18.3	20110306	14.0	20110803	24.7	20110609	19.7	20100717	28.3
20111205	18.2	20101119	18.3	20110307	14.0	20110804	24.6	20110610	19.8	20100718	28.2
20111205	18.2	20101119	18.4	20110307	14.0	20110804	24.6	20110610	19.8	20100718	28.0
20111205	18.2	20101119	18.3	20110307	14.0	20110804	24.5	20110610	20.2	20100718	27.5
20111205	18.3	20101119	18.4	20110307	13.9	20110804	24.7	20110610	20.5	20100718	26.8
20111205	18.3	20101119	18.5	20110307	14.3	20110804	25.2	20110610	20.9	20100718	27.3
20111205	18.3	20101119	18.4	20110307	14.3	20110804	25.4	20110610	21.0	20100718	28.0
20111205	18.2	20101119	18.4	20110307	14.1	20110804	25.0	20110610	20.5	20100718	28.4
20111205	18.2	20101119	18.3	20110307	13.7	20110804	24.8	20110610	20.2	20100718	28.5
20111206	18.2	20101120	18.3	20110308	13.6	20110805	24.6	20110611	20.1	20100719	27.0
20111206	18.2	20101120	18.3	20110308	13.4	20110805	24.7	20110611	20.3	20100719	27.1
20111206	18.1	20101120	18.2	20110308	13.5	20110805	24.7	20110611	20.4	20100719	25.9
20111206	18.1	20101120	18.2	20110308	13.7	20110805	25.0	20110611	20.5	20100719	25.9
20111206	18.1	20101120	18.5	20110308	14.0	20110805	25.5	20110611	21.3	20100719	25.6
20111206	18.0	20101120	18.5	20110308	14.1	20110805	25.8	20110611	21.4	20100719	27.1
20111206	18.0	20101120	18.4	20110308	13.8	20110805	25.2	20110611	20.8	20100719	27.4
20111206	18.0	20101120	18.3	20110308	13.7	20110805	24.8	20110611	19.6	20100719	27.3
20111207	18.0	20101121	18.2	20110309	13.5	20110806	24.6	20110612	19.5	20100720	25.5
20111207	17.9	20101121	18.1	20110309	13.4	20110806	24.6	20110612	20.2	20100720	25.3
20111207	17.9	20101121	18.1	20110309	13.6	20110806	24.6	20110612	20.7	20100720	25.2
20111207	17.9	20101121	18.1	20110309	13.7	20110806	25.1	20110612	20.7	20100720	25.1
20111207	17.9	20101121	18.4	20110309	14.3	20110806	25.7	20110612	21.3	20100720	26.1
20111207	17.9	20101121	18.4	20110309	14.5	20110806	25.7	20110612	21.3	20100720	27.0
20111207	17.9	20101121	18.2	20110309	14.2	20110806	25.2	20110612	20.5	20100720	27.8
20111207	17.9	20101121	18.1	20110309	14.0	20110806	25.0	20110612	20.4	20100720	27.8
20111208	17.9	20101122	18.1	20110310	13.8	20110807	25.1	20110613	19.9	20100721	26.0
20111208	17.8	20101122	18.2	20110310	13.7	20110807	25.0	20110613	20.3	20100721	26.0
20111208	17.8	20101122	18.1	20110310	13.7	20110807	25.0	20110613	20.5	20100721	25.8
20111208	17.8	20101122	18.3	20110310	13.7	20110807	25.4	20110613	21.0	20100721	25.3
20111208	17.8	20101122	18.4	20110310	14.2	20110807	26.1	20110613	21.4	20100721	26.1
20111208	17.7	20101122	18.3	20110310	14.4	20110807	26.2	20110613	21.4	20100721	27.2
20111208	17.8	20101122	18.3	20110310	14.1	20110807	25.7	20110613	21.0	20100721	28.0
20111208	17.8	20101122	18.3	20110310	13.9	20110807	25.8	20110613	20.7	20100721	28.4
20111209	17.8	20101123	18.3	20110311	13.7	20110808	25.7	20110614	20.6	20100722	27.6
20111209	17.8	20101123	18.3	20110311	13.7	20110808	25.5	20110614	20.7	20100722	25.9
20111209	17.7	20101123	18.3	20110311	13.7	20110808	25.4	20110614	20.8	20100722	25.4
20111209	17.7	20101123	18.2	20110311	13.7	20110808	25.7	20110614	21.1	20100722	25.2
20111209	17.9	20101123	18.2	20110311	14.6	20110808	26.4	20110614	21.5	20100722	26.6
20111209	17.9	20101123	18.1	20110311	14.8	20110808	26.5	20110614	21.6	20100722	27.5
20111209	17.8	20101123	18.2	20110311	14.5	20110808	26.3	20110614	21.2	20100722	28.0
20111209	17.7	20101123	18.1	20110311	14.2	20110808	26.1	20110614	21.1	20100722	28.1
20111210	17.6	20101124	18.1	20110312	14.1	20110809	25.9	20110615	20.9	20100723	28.0
20111210	17.6	20101124	18.1	20110312	13.9	20110809	25.8	20110615	20.8	20100723	25.8

20111210	17.5	20101124	18.0	20110312	13.8	20110809	25.7	20110615	20.9	20100723	25.2
20111210	17.6	20101124	18.0	20110312	14.0	20110809	25.9	20110615	21.0	20100723	25.6
20111210	17.8	20101124	18.0	20110312	14.4	20110809	26.7	20110615	21.9	20100723	25.9
20111210	17.8	20101124	17.9	20110312	14.5	20110809	26.6	20110615	22.3	20100723	27.3
20111210	17.6	20101124	17.9	20110312	14.2	20110809	26.0	20110615	21.6	20100723	27.7
20111210	17.4	20101124	17.8	20110312	13.9	20110809	25.5	20110615	21.3	20100723	28.0
20111211	17.3	20101125	17.7	20110313	13.8	20110810	24.9	20110616	21.3	20100724	28.0
20111211	17.4	20101125	17.7	20110313	13.7	20110810	24.9	20110616	21.2	20100724	27.3
20111211	17.4	20101125	17.7	20110313	13.7	20110810	25.2	20110616	21.2	20100724	27.3
20111211	17.5	20101125	17.7	20110313	13.7	20110810	25.4	20110616	21.3	20100724	26.7
20111211	17.6	20101125	17.7	20110313	13.8	20110810	25.7	20110616	22.2	20100724	27.0
20111211	17.6	20101125	17.8	20110313	13.9	20110810	25.6	20110616	22.5	20100724	27.4
20111211	17.5	20101125	17.7	20110313	13.9	20110810	25.3	20110616	21.9	20100724	27.2
20111211	17.3	20101125	17.7	20110313	13.9	20110810	25.1	20110616	21.5	20100724	27.3
20111212	17.3	20101126	17.6	20110314	13.9	20110811	24.7	20110617	21.4	20100725	26.8
20111212	17.4	20101126	17.6	20110314	13.8	20110811	24.7	20110617	21.3	20100725	25.8
20111212	17.4	20101126	17.7	20110314	13.8	20110811	24.8	20110617	21.4	20100725	25.3
20111212	17.6	20101126	17.6	20110314	14.0	20110811	25.1	20110617	22.0	20100725	24.6
20111212	17.9	20101126	17.7	20110314	14.5	20110811	25.6	20110617	22.8	20100725	24.5
20111212	17.9	20101126	17.7	20110314	14.6	20110811	25.7	20110617	23.0	20100725	26.0
20111212	17.8	20101126	17.7	20110314	14.4	20110811	25.2	20110617	22.4	20100725	26.2
20111212	17.8	20101127	17.6	20110314	14.3	20110811	24.9	20110617	22.2	20100725	25.9
20111213	17.7	20101127	17.6	20110315	14.1	20110812	24.7	20110618	22.1	20100726	25.4
20111213	17.7	20101127	17.6	20110315	13.9	20110812	24.6	20110618	22.0	20100726	25.1
20111213	17.6	20101127	17.6	20110315	13.8	20110812	24.7	20110618	22.1	20100726	23.8
20111213	17.5	20101127	17.5	20110315	14.0	20110812	25.0	20110618	22.4	20100726	23.9
20111213	17.5	20101128	17.4	20110315	14.7	20110812	25.6	20110618	23.3	20100726	24.0
20111213	17.5	20101128	17.3	20110315	14.8	20110812	25.7	20110618	23.7	20100726	24.5
20111213	17.4	20101128	17.3	20110315	14.6	20110812	25.3	20110618	23.7	20100726	24.6
20111213	17.5	20101128	17.5	20110315	14.5	20110812	25.1	20110618	23.6	20100726	24.9
20111214	17.6	20101128	17.5	20110316	14.4	20110813	24.8	20110619	23.4	20100727	24.8
20111214	17.5	20101128	17.6	20110316	14.3	20110813	24.7	20110619	23.1	20100727	24.7
20111214	17.4	20101128	17.6	20110316	14.2	20110813	24.7	20110619	22.8	20100727	23.5
20111214	17.4	20101128	17.8	20110316	14.3	20110813	25.0	20110619	23.2	20100727	23.6
20111214	17.8	20101129	17.8	20110316	14.8	20110813	25.7	20110619	23.8	20100727	24.5
20111214	17.9	20101129	17.6	20110316	14.8	20110813	25.8	20110619	23.5	20100727	24.8
20111214	17.9	20101129	17.6	20110316	14.4	20110813	25.4	20110619	22.5	20100727	25.1
20111214	18.0	20101129	17.5	20110316	14.2	20110813	25.1	20110619	21.4	20100727	24.8
20111215	18.0	20101129	17.6	20110317	14.3	20110814	25.0	20110620	21.0	20100728	24.1
20111215	18.0	20101129	17.5	20110317	14.3	20110814	24.8	20110620	21.0	20100728	23.1
20111215	18.0	20101129	17.5	20110317	14.2	20110814	24.8	20110620	21.3	20100728	23.5
20111215	18.0	20101129	17.5	20110317	14.3	20110814	24.9	20110620	22.3	20100728	23.9
20111215	18.0	20101130	17.3	20110317	14.8	20110814	25.5	20110620	22.7	20100728	24.6
20111215	18.0	20101130	17.2	20110317	14.8	20110814	25.7	20110620	22.6	20100728	25.2
20111215	17.9	20101130	17.2	20110317	14.5	20110814	25.3	20110620	22.4	20100728	25.7
20111215	17.8	20101130	17.3	20110317	14.6	20110814	25.0	20110620	22.2	20100728	25.6
20111216	17.7	20101130	17.5	20110318	14.5	20110815	24.8	20110621	22.0	20100729	25.6
20111216	17.4	20101130	17.5	20110318	14.4	20110815	24.6	20110621	22.0	20100729	25.5
20111216	17.5	20101130	17.4	20110318	14.4	20110815	24.5	20110621	22.1	20100729	25.3
20111216	17.4	20101130	17.4	20110318	14.3	20110815	24.8	20110621	22.4	20100729	25.2
20111216	17.4	20101201	17.4	20110318	14.4	20110815	25.7	20110621	23.1	20100729	25.3
20111216	17.4	20101201	17.3	20110318	14.4	20110815	25.7	20110621	23.1	20100729	25.7
20111216	17.5	20101201	17.2	20110318	14.3	20110815	25.3	20110621	22.3	20100729	26.1
20111216	17.6	20101201	17.3	20110318	14.3	20110815	25.2	20110621	21.9	20100729	26.0
20111217	17.6	20101201	17.4	20110319	14.3	20110816	25.0	20110622	22.9	20100730	25.9
20111217	17.5	20101201	17.3	20110319	14.4	20110816	24.9	20110622	23.0	20100730	25.3
20111217	17.3	20101201	17.5	20110319	14.2	20110816	24.8	20110622	22.9	20100730	24.9
20111217	17.2	20101201	17.5	20110319	14.3	20110816	25.2	20110622	23.1	20100730	24.9
20111217	17.2	20101202	17.4	20110319	14.7	20110816	25.8	20110622	23.0	20100730	25.0
20111217	17.1	20101202	17.4	20110319	14.9	20110816	25.9	20110622	23.5	20100730	24.8
20111217	17.3	20101202	17.3	20110319	14.6	20110816	25.5	20110622	23.4	20100730	25.2
20111217	17.4	20101202	17.4	20110319	14.4	20110816	25.4	20110622	23.5	20100730	25.2
20111218	17.3	20101202	17.5	20110320	14.3	20110817	25.3	20110623	23.5	20100731	25.2
20111218	17.5	20101202	17.4	20110320	14.3	20110817	25.1	20110623	23.4	20100731	25.2
20111218	17.7	20101202	17.4	20110320	14.2	20110817	25.0	20110623	23.2	20100731	25.1
20111218	17.7	20101202	17.4	20110320	14.3	20110817	25.3	20110623	23.4	20100731	25.2
20111218	17.7	20101203	17.3	20110320	14.5	20110817	25.8	20110623	23.5	20100731	25.3
20111218	17.8	20101203	17.3	20110320	14.8	20110817	26.1	20110623	24.0	20100731	25.5
20111218	17.7	20101203	17.3	20110320	14.6	20110817	25.7	20110623	23.7	20100731	25.6
20111218	17.7	20101203	17.3	20110320	14.5	20110817	25.5	20110623	23.4	20100731	25.5
20111219	17.6	20101203	17.3	20110321	14.4	20110818	25.4	20110624	23.2	20100801	25.4
20111219	17.6	20101203	17.2	20110321	14.3	20110818	25.1	20110624	22.9	20100801	25.3
20111219	17.3	20101203	17.2	20110321	14.3	20110818	25.2	20110624	22.7	20100801	25.3
20111219	17.0	20101203	17.1	20110321	14.2	20110818	25.6	20110624	23.1	20100801	25.2
20111219	17.0	20101204	17.2	20110321	14.8	20110818	26.2	20110624	24.0	20100801	25.4
20111219	17.0	20101204	17.1	20110321	15.0	20110818	26.3	20110624	24.3	20100801	26.0
20111219	16.9	20101204	17.0	20110321	14.7	20110818	25.8	20110624	23.5	20100801	26.0
20111219	17.0	20101204	17.0	20110321	14.5	20110818	25.6	20110624	23.2	20100801	26.0

20111220	17.0	20101204	17.0	20110322	14.3	20110819	25.4	20110625	22.8	20100802	25.9
20111220	17.3	20101204	17.0	20110322	14.2	20110819	25.2	20110625	23.4	20100802	25.6
20111220	17.2	20101204	17.0	20110322	14.3	20110819	25.4	20110625	23.3	20100802	25.5
20111220	17.3	20101204	17.0	20110322	14.4	20110819	25.7	20110625	23.5	20100802	25.5
20111220	17.4	20101205	17.0	20110322	14.8	20110819	26.4	20110625	23.8	20100802	25.6
20111220	17.3	20101205	16.9	20110322	15.2	20110819	26.5	20110625	23.6	20100802	26.0
20111220	17.2	20101205	16.9	20110322	14.7	20110819	25.9	20110625	23.3	20100802	26.4
20111220	17.3	20101205	16.9	20110322	14.6	20110819	25.7	20110625	23.0	20100802	26.3
20111221	17.2	20101205	17.0	20110323	14.5	20110820	25.5	20110626	23.1	20100803	25.9
20111221	17.0	20101205	17.0	20110323	14.4	20110820	25.5	20110626	22.9	20100803	25.0
20111221	16.9	20101205	16.9	20110323	14.4	20110820	25.6	20110626	22.6	20100803	25.4
20111221	17.1	20101205	16.8	20110323	14.6	20110820	25.8	20110626	22.7	20100803	25.0
20111221	17.0	20101206	16.8	20110323	15.1	20110820	26.5	20110626	23.5	20100803	25.4
20111221	17.0	20101206	16.8	20110323	15.5	20110820	26.5	20110626	23.9	20100803	25.7
20111221	16.9	20101206	16.6	20110323	14.9	20110820	26.0	20110626	23.6	20100803	22.2
20111221	16.8	20101206	16.7	20110323	14.5	20110820	25.8	20110626	23.5	20100803	22.1
20111222	16.6	20101206	17.0	20110324	14.4	20110821	25.9	20110627	23.2	20100804	25.9
20111222	16.4	20101206	17.0	20110324	14.3	20110821	25.9	20110627	22.8	20100804	25.8
20111222	16.2	20101206	16.9	20110324	14.2	20110821	25.9	20110627	22.4	20100804	24.9
20111222	16.2	20101206	16.8	20110324	14.4	20110821	26.2	20110627	23.1	20100804	25.7
20111222	16.4	20101207	16.7	20110324	15.2	20110821	26.8	20110627	23.9	20100804	26.0
20111222	16.5	20101207	16.7	20110324	15.2	20110821	26.8	20110627	24.1	20100804	26.3
20111222	16.4	20101207	16.8	20110324	14.9	20110821	26.3	20110627	23.6	20100804	26.3
20111222	16.4	20101207	16.8	20110324	14.7	20110821	26.1	20110627	23.5	20100804	26.4
20111223	16.4	20101207	17.1	20110325	14.5	20110822	26.1	20110628	23.2	20100805	26.2
20111223	16.3	20101207	17.1	20110325	14.4	20110822	26.0	20110628	23.1	20100805	25.9
20111223	16.3	20101207	17.0	20110325	14.4	20110822	25.9	20110628	23.1	20100805	24.6
20111223	16.4	20101207	17.0	20110325	14.6	20110822	26.1	20110628	23.4	20100805	25.4
20111223	16.7	20101208	16.8	20110325	15.6	20110822	26.8	20110628	23.9	20100805	25.8
20111223	16.7	20101208	16.7	20110325	15.9	20110822	26.7	20110628	24.1	20100805	26.1
20111223	16.6	20101208	16.6	20110325	15.2	20110822	26.3	20110628	23.6	20100805	26.6
20111223	16.5	20101208	16.8	20110325	14.7	20110822	26.2	20110628	23.2	20100805	26.4
20111224	16.6	20101208	17.0	20110326	14.4	20110823	25.9	20110629	23.1	20100806	26.3
20111224	16.7	20101208	17.1	20110326	14.3	20110823	25.7	20110629	23.0	20100806	26.1
20111224	16.5	20101208	17.0	20110326	14.3	20110823	25.7	20110629	23.0	20100806	25.7
20111224	16.5	20101208	17.0	20110326	14.6	20110823	26.1	20110629	23.4	20100806	25.6
20111224	16.8	20101209	16.9	20110326	15.2	20110823	26.8	20110629	23.3	20100806	25.7
20111224	17.0	20101209	16.9	20110326	15.6	20110823	27.3	20110629	23.5	20100806	25.7
20111224	17.0	20101209	16.9	20110326	15.1	20110823	27.0	20110629	23.5	20100806	25.7
20111224	16.9	20101209	16.9	20110326	14.9	20110823	26.8	20110629	23.4	20100806	25.7
20111225	17.0	20101209	17.0	20110327	14.8	20110824	26.7	20110630	23.3	20100807	25.5
20111225	17.0	20101209	16.9	20110327	14.8	20110824	26.6	20110630	23.1	20100807	25.4
20111225	17.0	20101209	16.9	20110327	14.8	20110824	26.5	20110630	23.2	20100807	24.9
20111225	17.0	20101209	16.8	20110327	14.9	20110824	26.7	20110630	23.6	20100807	24.0
20111225	17.0	20101210	16.8	20110327	15.7	20110824	27.2	20110630	24.2	20100807	24.6
20111225	17.0	20101210	16.8	20110327	16.1	20110824	27.4	20110630	24.5	20100807	25.3
20111225	17.0	20101210	16.8	20110327	15.9	20110824	26.6	20110630	24.0	20100807	25.4
20111225	16.9	20101210	16.7	20110327	15.7	20110824	26.2	20110630	23.6	20100807	25.3
20111226	16.9	20101210	16.6	20110328	15.5	20110825	26.3	20110701	23.4	20100808	25.2
20111226	16.9	20101210	16.5	20110328	15.3	20110825	26.2	20110701	23.1	20100808	25.0
20111226	16.9	20101210	16.6	20110328	15.2	20110825	26.3	20110701	23.0	20100808	24.3
20111226	16.9	20101210	16.6	20110328	15.3	20110825	26.5	20110701	23.5	20100808	24.8
20111226	17.0	20101211	16.4	20110328	15.5	20110825	27.3	20110701	24.1	20100808	24.9
20111226	17.0	20101211	16.4	20110328	15.8	20110825	27.5	20110701	24.1	20100808	25.3
20111226	16.9	20101211	16.3	20110328	15.6	20110825	26.8	20110701	23.5	20100808	25.8
20111226	16.7	20101211	16.3	20110328	15.3	20110825	26.8	20110701	23.2	20100808	25.7
20111227	16.6	20101211	16.3	20110329	14.9	20110826	26.2	20110702	23.0	20100809	25.6
20111227	16.5	20101211	16.2	20110329	14.8	20110826	26.1	20110702	23.3	20100809	25.5
20111227	16.4	20101211	16.1	20110329	14.7	20110826	26.3	20110702	23.6	20100809	25.4
20111227	16.4	20101211	16.2	20110329	14.8	20110826	26.7	20110702	23.9	20100809	25.0
20111227	16.5	20101212	16.2	20110329	15.2	20110826	27.3	20110702	24.5	20100809	25.6
20111227	16.3	20101212	16.3	20110329	15.2	20110826	27.3	20110702	24.3	20100809	25.7
20111227	16.3	20101212	16.3	20110329	14.8	20110826	27.0	20110702	24.1	20100809	26.3
20111227	16.0	20101212	16.4	20110329	14.7	20110826	26.9	20110702	24.0	20100809	26.3
20111228	16.1	20101212	16.4	20110330	14.7	20110827	26.9	20110703	23.5	20100810	26.3
20111228	16.1	20101212	16.4	20110330	14.7	20110827	26.8	20110703	23.5	20100810	25.8
20111228	16.0	20101212	16.4	20110330	14.6	20110827	26.8	20110703	23.4	20100810	25.6
20111228	16.2	20101212	16.5	20110330	14.8	20110827	26.9	20110703	23.5	20100810	24.8
20111228	16.3	20101213	16.5	20110330	15.4	20110827	27.6	20110703	24.6	20100810	25.7
20111228	16.3	20101213	16.5	20110330	15.7	20110827	27.8	20110703	24.4	20100810	26.2
20111228	16.2	20101213	16.4	20110330	15.2	20110827	27.2	20110703	24.0	20100810	26.5
20111228	16.2	20101213	16.4	20110330	14.8	20110827	27.0	20110703	23.7	20100810	26.6
20111229	16.2	20101213	16.4	20110331	14.7	20110828	26.8	20110704	23.5	20100811	26.3
20111229	16.1	20101213	16.3	20110331	14.7	20110828	26.0	20110704	23.4	20100811	25.9
20111229	16.1	20101213	16.4	20110331	14.7	20110828	25.0	20110704	23.5	20100811	25.9
20111229	16.2	20101213	16.4	20110331	14.8	20110828	24.9	20110704	24.1	20100811	25.9
20111229	16.6	20101214	16.2	20110331	15.3	20110828	25.5	20110704	24.7	20100811	26.1
20111229	16.6	20101214	16.3	20110331	15.6	20110828	25.4	20110704	24.7	20100811	26.4

20111229	16.4	20101214	16.3	20110331	15.2	20110828	25.9	20110704	24.4	20100811	26.6
20111229	16.3	20101214	16.3	20110331	14.8	20110828	25.7	20110704	24.1	20100811	26.6
20111230	16.3	20101214	16.4	20110401	14.8	20110829	25.7	20110705	23.9	20100812	26.6
20111230	16.3	20101214	16.3	20110401	14.7	20110829	25.7	20110705	23.9	20100812	26.3
20111230	16.3	20101214	16.2	20110401	14.6	20110829	26.0	20110705	23.9	20100812	25.9
20111230	16.3	20101214	16.1	20110401	14.8	20110829	26.4	20110705	24.1	20100812	25.4
20111230	16.6	20101215	15.9	20110401	15.2	20110829	26.3	20110705	24.3	20100812	25.9
20111230	16.6	20101215	16.0	20110401	15.3	20110829	25.8	20110705	24.2	20100812	26.4
20111230	16.6	20101215	16.0	20110401	15.1	20110829	25.7	20110705	23.9	20100812	26.8
20111230	16.5	20101215	15.6	20110401	14.8	20110830	25.4	20110705	23.6	20100812	26.8
20111231	16.5	20101215	15.6	20110402	14.7	20110830	25.3	20110706	23.8	20100813	26.7
20111231	16.4	20101215	15.8	20110402	14.8	20110830	25.3	20110706	23.8	20100813	26.0
20111231	16.4	20101215	15.9	20110402	14.8	20110830	25.6	20110706	23.8	20100813	25.7
20111231	16.3	20101215	15.8	20110402	15.1	20110830	26.0	20110706	23.9	20100813	25.7
20111231	16.4	20101216	15.7	20110402	15.7	20110830	25.9	20110706	24.2	20100813	25.9
20111231	16.4	20101216	15.8	20110402	15.9	20110830	25.7	20110706	24.6	20100813	26.3
20111231	16.4	20101216	15.9	20110402	15.9	20110830	25.3	20110706	24.3	20100813	26.7
20111231	16.3	20101216	15.9	20110402	15.6	20110831	25.0	20110706	24.2	20100813	27.0
20120101	16.3	20101216	16.1	20110403	15.4	20110831	24.9	20110707	24.1	20100814	26.4
20120101	16.3	20101216	16.1	20110403	15.4	20110831	25.1	20110707	23.8	20100814	26.3
20120101	16.3	20101216	16.0	20110403	15.3	20110831	25.3	20110707	23.5	20100814	25.8
20120101	16.3	20101216	15.9	20110403	15.3	20110831	25.8	20110707	23.9	20100814	25.8
20120101	16.5	20101217	15.9	20110403	15.8	20110831	25.9	20110707	25.1	20100814	26.0
20120101	16.5	20101217	15.7	20110403	15.7	20110831	25.5	20110707	25.0	20100814	27.0
20120101	16.4	20101217	15.5	20110403	15.4	20110831	25.4	20110707	24.6	20100814	26.9
20120101	16.4	20101217	15.8	20110403	15.3	20110901	25.3	20110707	24.1	20100814	26.8
20120102	16.3	20101217	15.9	20110404	15.3	20110901	25.1	20110708	24.0	20100815	26.9
20120102	16.2	20101217	15.9	20110404	15.3	20110901	25.2	20110708	23.9	20100815	26.2
20120102	16.1	20101217	15.9	20110404	15.3	20110901	25.4	20110708	24.1	20100815	26.1
20120102	16.2	20101217	15.9	20110404	15.6	20110901	25.9	20110708	24.7	20100815	26.1
20120102	16.6	20101218	15.9	20110404	16.2	20110901	26.2	20110708	25.5	20100815	26.2
20120102	16.6	20101218	15.9	20110404	16.2	20110901	25.9	20110708	25.8	20100815	26.2
20120102	16.5	20101218	15.9	20110404	15.7	20110901	25.6	20110708	24.9	20100815	26.3
20120102	16.5	20101218	15.9	20110404	15.5	20110902	25.5	20110708	24.6	20100815	26.3
20120103	16.4	20101218	15.9	20110405	15.4	20110902	25.2	20110709	24.4	20100816	26.2
20120103	16.3	20101218	15.9	20110405	15.3	20110902	25.4	20110709	24.6	20100816	25.8
20120103	16.3	20101218	15.9	20110405	15.2	20110902	25.7	20110709	24.6	20100816	25.6
20120103	16.3	20101218	15.9	20110405	15.5	20110902	26.1	20110709	24.9	20100816	25.6
20120103	16.5	20101219	15.9	20110405	16.2	20110902	26.1	20110709	25.7	20100816	25.8
20120103	16.6	20101219	15.9	20110405	17.0	20110902	26.0	20110709	25.7	20100816	26.1
20120103	16.5	20101219	15.9	20110405	16.5	20110902	25.9	20110709	25.5	20100816	26.0
20120103	16.5	20101219	15.9	20110405	16.1	20110903	25.7	20110709	25.2	20100816	25.9
20120104	16.6	20101219	15.9	20110406	15.9	20110903	25.7	20110710	25.0	20100817	25.9
20120104	16.5	20101219	15.9	20110406	15.5	20110903	25.7	20110710	24.9	20100817	25.3
20120104	16.5	20101219	15.9	20110406	15.1	20110903	25.9	20110710	24.8	20100817	25.0
20120104	16.5	20101219	15.8	20110406	15.1	20110903	26.1	20110710	25.3	20100817	22.3
20120104	16.7	20101220	15.7	20110406	15.7	20110903	26.2	20110710	26.2	20100817	23.5
20120104	16.7	20101220	15.8	20110406	16.1	20110903	26.0	20110710	26.0	20100817	24.3
20120104	16.6	20101220	15.8	20110406	15.8	20110903	25.7	20110710	26.0	20100817	24.6
20120104	16.5	20101220	15.9	20110406	15.6	20110904	25.7	20110710	25.7	20100817	24.6
20120105	16.5	20101220	16.2	20110407	15.5	20110904	25.7	20110711	25.3	20100818	24.6
20120105	16.4	20101220	16.2	20110407	15.4	20110904	25.6	20110711	25.0	20100818	24.2
20120105	16.4	20101220	16.1	20110407	15.3	20110904	25.9	20110711	24.9	20100818	24.0
20120105	16.4	20101220	16.1	20110407	15.6	20110904	26.2	20110711	25.2	20100818	23.8
20120105	16.5	20101221	16.1	20110407	16.2	20110904	25.8	20110711	26.1	20100818	23.9
20120105	16.5	20101221	16.2	20110407	16.3	20110904	25.7	20110711	26.6	20100818	24.2
20120105	16.5	20101221	16.1	20110407	16.0	20110904	25.7	20110711	26.2	20100818	24.4
20120105	16.4	20101221	16.1	20110407	15.7	20110905	25.7	20110711	25.7	20100818	24.6
20120106	16.4	20101221	16.3	20110408	15.5	20110905	25.7	20110712	25.6	20100819	24.3
20120106	16.4	20101221	16.2	20110408	15.4	20110905	25.6	20110712	25.5	20100819	24.1
20120106	16.4	20101221	16.1	20110408	15.4	20110905	25.8	20110712	25.5	20100819	23.0
20120106	16.3	20101221	16.1	20110408	15.8	20110905	26.3	20110712	25.8	20100819	23.9
20120106	16.1	20101222	15.9	20110408	16.2	20110905	26.4	20110712	26.4	20100819	24.1
20120106	16.2	20101222	16.0	20110408	16.2	20110905	25.9	20110712	26.6	20100819	24.5
20120106	16.1	20101222	15.9	20110408	15.5	20110905	25.7	20110712	25.9	20100819	24.7
20120106	16.0	20101222	15.9	20110408	15.3	20110906	25.5	20110712	25.5	20100819	25.2
20120107	16.0	20101222	16.0	20110409	15.1	20110906	25.6	20110713	25.7	20100820	24.8
20120107	16.0	20101222	16.1	20110409	15.3	20110906	25.6	20110713	25.8	20100820	24.6
20120107	16.0	20101222	16.0	20110409	15.4	20110906	25.7	20110713	25.7	20100820	24.5
20120107	16.0	20101222	16.0	20110409	15.9	20110906	26.2	20110713	26.0	20100820	24.5
20120107	16.0	20101223	15.9	20110409	16.5	20110906	26.2	20110713	26.7	20100820	24.7
20120107	15.9	20101223	15.9	20110409	16.4	20110906	25.7	20110713	27.1	20100820	25.2
20120107	15.9	20101223	15.9	20110409	16.1	20110906	25.3	20110713	26.6	20100820	25.4
20120107	15.9	20101223	15.9	20110409	16.0	20110907	25.1	20110713	26.5	20100820	25.3
20120108	15.9	20101223	15.9	20110410	16.0	20110907	25.1	20110714	26.4	20100821	25.6
20120108	15.9	20101223	15.9	20110410	15.9	20110907	25.1	20110714	26.3	20100821	25.5
20120108	15.9	20101223	15.9	20110410	15.9	20110907	25.4	20110714	26.2	20100821	25.4
20120108	15.9	20101223	15.9	20110410	16.2	20110907	25.7	20110714	25.2	20100821	25.3

20120108	16.0	20101224	15.9	20110410	17.3	20110907	25.8	20110714	26.2	20100821	25.7
20120108	16.0	20101224	15.9	20110410	17.2	20110907	25.5	20110714	26.8	20100821	26.5
20120108	16.0	20101224	15.9	20110410	16.6	20110907	25.4	20110714	24.9	20100821	26.9
20120108	15.9	20101224	15.9	20110410	17.0	20110908	25.3	20110714	24.6	20100821	27.1
20120109	15.9	20101224	16.0	20110411	16.5	20110908	25.3	20110715	24.3	20100821	27.1
20120109	16.0	20101224	15.9	20110411	16.4	20110908	25.2	20110715	24.4	20100822	26.9
20120109	15.9	20101224	15.9	20110411	16.6	20110908	25.4	20110715	24.8	20100822	25.7
20120109	15.9	20101224	16.0	20110411	16.6	20110908	25.8	20110715	24.2	20100822	26.0
20120109	15.9	20101225	15.9	20110411	17.1	20110908	25.8	20110715	23.7	20100822	26.0
20120109	15.9	20101225	15.9	20110411	17.5	20110908	25.6	20110715	25.1	20100822	26.6
20120109	15.9	20101225	15.9	20110411	17.2	20110908	25.4	20110715	24.7	20100822	27.1
20120109	15.8	20101225	15.9	20110411	16.5	20110909	25.3	20110715	24.5	20100822	27.0
20120110	15.7	20101225	15.9	20110412	16.3	20110909	25.3	20110716	24.4	20100823	26.9
20120110	15.7	20101225	15.9	20110412	16.1	20110909	25.3	20110716	24.5	20100823	26.8
20120110	15.5	20101225	15.9	20110412	16.0	20110909	25.4	20110716	24.4	20100823	24.8
20120110	15.3	20101225	15.9	20110412	16.1	20110909	25.6	20110716	24.8	20100823	25.4
20120110	15.5	20101226	15.9	20110412	16.6	20110909	25.7	20110716	25.4	20100823	26.0
20120110	15.5	20101226	15.9	20110412	16.4	20110909	25.5	20110716	25.9	20100823	26.1
20120110	15.5	20101226	15.9	20110412	16.0	20110909	25.4	20110716	25.7	20100823	26.4
20120110	15.5	20101226	15.9	20110412	15.8	20110910	25.3	20110716	25.4	20100823	26.5
20120111	15.5	20101226	15.9	20110413	15.9	20110910	25.2	20110717	25.3	20100824	26.4
20120111	15.6	20101226	15.9	20110413	16.0	20110910	25.2	20110717	25.6	20100824	26.3
20120111	15.6	20101226	15.9	20110413	16.0	20110910	25.5	20110717	25.4	20100824	26.2
20120111	15.6	20101226	15.8	20110413	16.3	20110910	26.0	20110717	25.8	20100824	25.9
20120111	15.7	20101227	15.7	20110413	16.8	20110910	26.1	20110717	26.4	20100824	26.2
20120111	15.6	20101227	15.7	20110413	16.3	20110910	25.8	20110717	26.6	20100824	26.9
20120111	15.6	20101227	15.8	20110413	15.0	20110910	25.7	20110717	25.7	20100824	27.2
20120111	15.5	20101227	15.8	20110413	15.0	20110911	25.5	20110717	25.7	20100824	27.1
20120112	15.5	20101227	15.9	20110414	14.8	20110911	25.3	20110718	25.6	20100825	27.0
20120112	15.5	20101227	15.9	20110414	15.1	20110911	25.3	20110718	25.5	20100825	26.5
20120112	15.5	20101227	15.9	20110414	15.6	20110911	25.6	20110718	25.6	20100825	26.2
20120112	15.4	20101227	15.9	20110414	15.8	20110911	26.2	20110718	25.9	20100825	26.0
20120112	15.7	20101228	15.8	20110414	16.3	20110911	26.4	20110718	26.6	20100825	26.3
20120112	15.7	20101228	15.8	20110414	16.7	20110911	25.9	20110718	26.7	20100825	26.9
20120112	15.6	20101228	15.8	20110414	16.3	20110911	25.7	20110718	25.8	20100825	27.3
20120112	15.5	20101228	15.7	20110414	16.1	20110912	25.6	20110718	25.8	20100825	27.3
20120113	15.4	20101228	15.8	20110415	16.0	20110912	25.4	20110719	25.7	20100826	26.4
20120113	15.4	20101228	15.7	20110415	15.9	20110912	25.4	20110719	25.5	20100826	26.3
20120113	15.5	20101228	15.6	20110415	15.9	20110912	25.5	20110719	25.5	20100826	24.8
20120113	15.5	20101228	15.7	20110415	16.0	20110912	25.9	20110719	26.0	20100826	25.0
20120113	15.7	20101229	15.6	20110415	16.2	20110912	26.2	20110719	26.6	20100826	25.8
20120113	15.8	20101229	15.5	20110415	16.1	20110912	25.8	20110719	26.8	20100826	26.3
20120113	15.7	20101229	15.5	20110415	16.0	20110912	25.6	20110719	26.3	20100826	26.8
20120113	15.6	20101229	15.6	20110415	15.9	20110913	25.5	20110719	26.1	20100826	26.8
20120114	15.6	20101229	15.7	20110416	15.9	20110913	25.4	20110720	26.0	20100827	26.7
20120114	15.6	20101229	15.7	20110416	15.8	20110913	25.3	20110720	25.9	20100827	26.0
20120114	15.7	20101229	15.6	20110416	15.5	20110913	25.6	20110720	25.8	20100827	25.5
20120114	15.7	20101229	15.6	20110416	15.7	20110913	25.9	20110720	25.4	20100827	25.2
20120114	15.8	20101230	15.5	20110416	16.0	20110913	25.8	20110720	24.2	20100827	25.7
20120114	15.8	20101230	15.4	20110416	16.1	20110913	25.7	20110720	24.7	20100827	26.9
20120114	15.8	20101230	15.4	20110416	15.9	20110913	25.5	20110720	24.9	20100827	27.0
20120114	15.7	20101230	15.5	20110416	15.8	20110914	25.4	20110720	24.8	20100827	26.7
20120115	15.6	20101230	15.8	20110417	15.6	20110914	25.3	20110721	24.7	20100828	26.7
20120115	15.5	20101230	15.9	20110417	15.5	20110914	25.2	20110721	24.6	20100828	25.9
20120115	15.3	20101230	15.7	20110417	15.4	20110914	25.4	20110721	24.4	20100828	25.8
20120115	15.2	20101230	15.5	20110417	15.7	20110914	25.9	20110721	24.5	20100828	24.9
20120115	15.4	20101231	15.4	20110417	16.2	20110914	26.0	20110721	24.8	20100828	25.8
20120115	15.4	20101231	15.3	20110417	15.8	20110914	25.6	20110721	24.8	20100828	25.7
20120115	15.4	20101231	15.3	20110417	15.6	20110914	25.4	20110721	24.7	20100828	26.0
20120115	15.3	20101231	15.3	20110417	15.5	20110915	25.3	20110721	24.7	20100828	26.6
20120116	15.2	20101231	15.5	20110418	15.4	20110915	25.1	20110722	24.6	20100829	26.6
20120116	15.2	20101231	15.6	20110418	15.5	20110915	25.0	20110722	24.6	20100829	26.5
20120116	15.2	20101231	15.4	20110418	15.4	20110915	25.0	20110722	24.5	20100829	26.4
20120116	15.1	20101231	15.3	20110418	15.6	20110915	25.6	20110722	24.9	20100829	26.2
20120116	15.2	20110101	15.3	20110418	16.1	20110915	25.6	20110722	25.6	20100829	26.4
20120116	15.1	20110101	15.3	20110418	16.0	20110915	25.2	20110722	25.5	20100829	26.8
20120116	15.0	20110101	15.2	20110418	15.8	20110915	25.3	20110722	25.3	20100829	26.9
20120116	14.9	20110101	15.2	20110418	15.7	20110916	25.3	20110722	25.0	20100829	26.7
20120117	14.8	20110101	15.5	20110419	15.6	20110916	25.1	20110723	24.7	20100830	26.4
20120117	15.0	20110101	15.5	20110419	15.5	20110916	25.2	20110723	24.7	20100830	26.3
20120117	15.0	20110101	15.4	20110419	15.5	20110916	25.4	20110723	24.7	20100830	26.2
20120117	15.1	20110101	15.2	20110419	15.6	20110916	26.0	20110723	24.9	20100830	25.8
20120117	15.4	20110102	15.2	20110419	16.2	20110916	26.1	20110723	25.4	20100830	25.7
20120117	15.4	20110102	15.1	20110419	16.9	20110916	25.5	20110723	25.7	20100830	26.3
20120117	15.1	20110102	15.1	20110419	16.6	20110916	25.4	20110723	25.2	20100830	26.5
20120117	14.9	20110102	15.2	20110419	16.3	20110917	25.3	20110723	24.7	20100830	26.4
20120118	15.0	20110102	15.3	20110420	16.0	20110917	25.2	20110724	24.6	20100831	26.3
20120118	15.0	20110102	15.4	20110420	15.9	20110917	25.2	20110724	24.6	20100831	26.1

20120118	15.0
20120118	15.1
20120118	15.5
20120118	15.6
20120118	15.3
20120118	15.2
20120119	15.1
20120119	15.0
20120119	15.1
20120119	15.3
20120119	15.6
20120119	15.7
20120119	15.5
20120119	15.3
20120120	15.2
20120120	15.1
20120120	15.2
20120120	15.3
20120120	15.4
20120120	15.5
20120120	15.4
20120120	15.4
20120121	15.5
20120121	15.5
20120121	15.4
20120121	15.4
20120121	15.5
20120121	15.5
20120121	15.5
20120121	15.4
20120122	15.5
20120122	15.5
20120122	15.4
20120122	15.4
20120122	15.6
20120122	15.6
20120122	15.4
20120122	15.4
20120123	15.4
20120123	15.5
20120123	15.4
20120123	15.4
20120123	15.5
20120123	15.5
20120123	15.4
20120123	15.4
20120124	15.4
20120124	15.4
20120124	15.4
20120124	15.3
20120124	15.5
20120124	15.5
20120124	15.4
20120124	15.3
20120125	15.3
20120125	15.3
20120125	15.1
20120125	15.1
20120125	15.2
20120125	15.2
20120125	15.1
20120125	14.8
20120126	14.8
20120126	14.8
20120126	14.8
20120126	14.8
20120126	15.0
20120126	15.1
20120126	14.9
20120126	14.8
20120127	14.8
20120127	14.8
20120127	14.8
20120127	14.9
20120127	15.2
20120127	15.3
20120127	15.0
20120127	14.9

20110102	15.3
20110102	15.1
20110103	15.0
20110103	15.1
20110103	15.0
20110103	15.2
20110103	15.3
20110103	15.3
20110103	15.2
20110103	15.3
20110104	15.2
20110104	15.3
20110104	15.2
20110104	15.2
20110104	15.4
20110104	15.5
20110104	15.4
20110104	15.3
20110105	15.2
20110105	15.2
20110105	15.2
20110105	15.1
20110105	15.4
20110105	15.4
20110105	15.3
20110105	15.1
20110106	14.9
20110106	15.0
20110106	15.0
20110106	15.1
20110106	15.4
20110106	15.5
20110106	15.3
20110106	15.2
20110107	15.2
20110107	15.2
20110107	15.1
20110107	15.1
20110107	15.5
20110107	15.7
20110107	15.6
20110107	15.4
20110108	15.3
20110108	15.2
20110108	15.1
20110108	15.1
20110108	15.4
20110108	15.5
20110108	15.3
20110108	15.2
20110109	15.1
20110109	15.0
20110109	15.0
20110109	15.0
20110109	15.3
20110109	15.6
20110109	15.4
20110109	15.3
20110110	15.1
20110110	15.0
20110110	15.0
20110110	15.0
20110110	15.3
20110110	15.5
20110110	15.3
20110110	15.2
20110111	15.0
20110111	15.1
20110111	15.1
20110111	15.2
20110111	15.4
20110111	15.4
20110111	15.2
20110111	15.2
20110112	15.2
20110112	15.1
20110112	15.0
20110112	15.1

20110420	15.8
20110420	16.0
20110420	17.0
20110420	17.3
20110420	16.6
20110420	16.1
20110421	15.9
20110421	15.9
20110421	16.0
20110421	16.4
20110421	17.3
20110421	17.5
20110421	17.2
20110421	16.9
20110422	16.6
20110422	16.5
20110422	16.4
20110422	16.8
20110422	17.1
20110422	17.0
20110422	16.9
20110422	16.7
20110423	16.5
20110423	16.2
20110423	16.2
20110423	16.5
20110423	17.0
20110423	17.5
20110423	17.3
20110423	17.2
20110424	17.0
20110424	16.6
20110424	16.6
20110424	17.0
20110424	18.3
20110424	18.3
20110424	17.8
20110424	16.9
20110425	16.5
20110425	16.3
20110425	16.0
20110425	16.1
20110425	16.4
20110425	16.2
20110425	15.9
20110425	15.8
20110426	15.6
20110426	15.4
20110426	15.4
20110426	15.6
20110426	16.0
20110426	15.8
20110426	15.5
20110426	15.2
20110427	15.3
20110427	15.4
20110427	15.5
20110427	15.8
20110427	15.9
20110427	15.8
20110427	15.5
20110427	15.4
20110428	15.3
20110428	15.3
20110428	15.5
20110428	15.5
20110428	15.9
20110428	16.1
20110428	16.1
20110428	15.9
20110429	15.8
20110429	15.8
20110429	15.8
20110429	16.0
20110429	16.8
20110429	16.9
20110429	16.4
20110429	16.2

20110917	25.4
20110917	32.5
20110917	25.6
20110917	25.4
20110918	25.2
20110918	25.2
20110918	25.2
20110918	25.4
20110918	26.2
20110918	26.0
20110918	25.8
20110918	25.6
20110919	25.5
20110919	25.2
20110919	25.2
20110919	25.4
20110919	24.7
20110919	25.0
20110919	24.8
20110919	25.0
20110920	25.0
20110920	25.0
20110920	24.8
20110920	24.6
20110920	24.7
20110920	24.6
20110920	24.1
20110920	24.2
20110921	24.3
20110921	24.2
20110921	24.2
20110921	24.1
20110921	24.1
20110921	24.1
20110921	23.7
20110921	23.7
20110922	23.8
20110922	23.8
20110922	23.8
20110922	24.0
20110922	24.6
20110922	24.6
20110922	24.4
20110922	24.2
20110923	24.1
20110923	24.0
20110923	24.0
20110923	24.3
20110923	24.7
20110923	24.7
20110923	24.4
20110923	24.2
20110924	24.0
20110924	23.9
20110924	23.9
20110924	24.2
20110924	24.7
20110924	24.7
20110924	24.4
20110924	24.2
20110925	24.0
20110925	23.9
20110925	24.0
20110925	24.1
20110925	24.4
20110925	24.3
20110925	24.2
20110925	24.2
20110926	24.1
20110926	24.1
20110926	23.9
20110926	23.9
20110926	24.0
20110926	24.0
20110926	24.0
20110926	24.0
20110927	23.9
20110927	23.9

20110724	24.6
20110724	24.8
20110724	25.3
20110724	25.2
20110724	24.3
20110724	24.3
20110725	24.4
20110725	24.4
20110725	24.5
20110725	24.3
20110725	23.9
20110725	24.0
20110725	24.0
20110725	24.3
20110726	24.3
20110726	24.3
20110726	24.3
20110726	24.4
20110726	24.7
20110726	24.9
20110726	24.7
20110726	24.6

20100831	25.5
20100831	25.1
20100831	25.1
20100831	25.8
20100831	25.8
20100831	25.9
20100901	25.7
20100901	25.6
20100901	25.1
20100901	24.8
20100901	25.2
20100901	25.6
20100901	25.5
20100901	25.6
20100902	25.5
20100902	25.5
20100902	25.5
20100902	25.5
20100902	25.6
20100902	25.5
20100902	25.6
20100902	25.4
20100903	25.3
20100903	25.2
20100903	25.1
20100903	24.5
20100903	24.8
20100903	24.9
20100903	24.7
20100903	24.7
20100904	24.7
20100904	24.6
20100904	24.5
20100904	24.5
20100904	24.6
20100904	24.7
20100904	24.5
20100904	24.5
20100905	24.4
20100905	24.0
20100905	23.8
20100905	23.7
20100905	23.7
20100905	24.3
20100905	24.5
20100905	24.8
20100906	24.6
20100906	24.5
20100906	24.3
20100906	24.3
20100906	24.4
20100906	24.6
20100906	25.3
20100906	25.2
20100907	25.2
20100907	24.7
20100907	24.3
20100907	24.4
20100907	24.9
20100907	25.2
20100907	25.0
20100907	24.6
20100908	24.7
20100908	24.5
20100908	24.5
20100908	24.5
20100908	24.6
20100908	24.6
20100908	24.5
20100908	24.5
20100909	24.5
20100909	24.5
20100909	24.5
20100909	24.5
20100909	24.4
20100909	24.5
20100909	24.5
20100909	24.6

20120128	14.8
20120128	14.8
20120128	14.8
20120128	14.8
20120128	15.0
20120128	15.1
20120128	14.9
20120128	14.8
20120129	14.8
20120129	14.8
20120129	14.6
20120129	14.6
20120129	14.9
20120129	14.9
20120129	14.8
20120129	14.8
20120130	14.7
20120130	14.7
20120130	14.8
20120130	14.8
20120130	14.8
20120130	14.8
20120130	14.6
20120130	14.6
20120131	14.6
20120131	14.5
20120131	14.4
20120131	14.6
20120131	14.8
20120131	14.8
20120131	14.8
20120131	14.8
20120201	14.8
20120201	14.6
20120201	14.6
20120201	14.8
20120201	14.8
20120201	14.8
20120201	14.8
20120201	14.8
20120202	14.8
20120202	14.8
20120202	14.8
20120202	14.8
20120202	14.9
20120202	14.8
20120202	14.8
20120202	14.7
20120203	14.7
20120203	14.6
20120203	14.6
20120203	14.5
20120203	14.6
20120203	14.6
20120203	14.6
20120203	14.5
20120204	14.6
20120204	14.6
20120204	14.6
20120204	14.6
20120204	14.7
20120204	14.8
20120204	14.7
20120204	14.7
20120205	14.7
20120205	14.6
20120205	14.4
20120205	14.5
20120205	14.7
20120205	14.7
20120205	14.5
20120205	14.4
20120206	14.4
20120206	14.5
20120206	14.5
20120206	14.4
20120206	14.5
20120206	14.5

20110112	15.3
20110112	15.3
20110112	15.2
20110112	15.2
20110113	15.1
20110113	15.1
20110113	15.0
20110113	15.1
20110113	15.3
20110113	15.3
20110113	15.1
20110113	15.1
20110114	15.1
20110114	15.0
20110114	14.9
20110114	15.0
20110114	15.3
20110114	15.3
20110114	15.2
20110114	15.1
20110115	15.1
20110115	15.0
20110115	14.9
20110115	15.0
20110115	15.3
20110115	15.3
20110115	15.2
20110115	15.1
20110116	15.1
20110116	15.0
20110116	14.9
20110116	14.9
20110116	15.3
20110116	15.4
20110116	15.2
20110116	15.1
20110117	15.1
20110117	15.1
20110117	15.1
20110117	15.1
20110117	15.4
20110117	15.4
20110117	15.3
20110117	15.2
20110118	15.2
20110118	15.2
20110118	15.0
20110118	15.0
20110118	15.4
20110118	15.5
20110118	15.3
20110118	15.2
20110119	15.1
20110119	15.1
20110119	15.0
20110119	15.0
20110119	15.4
20110119	15.4
20110119	15.3
20110119	15.1
20110120	15.0
20110120	15.1
20110120	15.0
20110120	14.9
20110120	15.2
20110120	15.2
20110120	15.1
20110120	14.9
20110121	14.9
20110121	14.9
20110121	14.9
20110121	14.9
20110121	15.1
20110121	15.1
20110121	15.1
20110121	15.0
20110122	14.9
20110122	14.9

20110430	15.9
20110430	15.8
20110430	15.8
20110430	15.8
20110430	15.9
20110430	16.1
20110430	16.1
20110430	16.1
20110501	16.1
20110501	16.2
20110501	16.2
20110501	16.3
20110501	16.3
20110501	16.4
20110501	16.1
20110501	16.0
20110502	15.9
20110502	16.2
20110502	16.3
20110502	16.5
20110502	16.7
20110502	16.5
20110502	16.3
20110502	16.0
20110503	16.1
20110503	16.2
20110503	16.2
20110503	16.5
20110503	16.3
20110503	16.3
20110503	16.2
20110503	16.1
20110504	16.1
20110504	16.2
20110504	16.2
20110504	16.2
20110504	16.2
20110504	16.4
20110504	16.1
20110504	16.0
20110505	16.0
20110505	16.0
20110505	16.1
20110505	16.3
20110505	16.8
20110505	17.0
20110505	16.7
20110505	16.4
20110506	16.3
20110506	16.3
20110506	16.3
20110506	16.8
20110506	17.5
20110506	17.3
20110506	17.8
20110506	17.0
20110507	16.6
20110507	16.6
20110507	16.8
20110507	17.0
20110507	18.1
20110507	18.1
20110507	17.1
20110507	16.9
20110508	16.7
20110508	16.6
20110508	16.6
20110508	17.0
20110508	18.1
20110508	18.2
20110508	18.1
20110508	17.1
20110509	16.8
20110509	17.0
20110509	17.1
20110509	17.3
20110509	17.5
20110509	17.2

20110927	23.6
20110927	23.7
20110927	24.0
20110927	24.1
20110927	23.9
20110927	23.7
20110928	23.5
20110928	23.4
20110928	23.5
20110928	23.6
20110928	24.1
20110928	24.1
20110928	23.8
20110928	23.6
20110929	23.5
20110929	23.3
20110929	23.3
20110929	23.5
20110929	23.9
20110929	24.2
20110929	23.6
20110929	23.5
20110930	23.5
20110930	23.2
20110930	23.0
20110930	23.5
20110930	24.0
20110930	24.0
20110930	23.4
20110930	23.3
20111001	23.2
20111001	23.2
20111001	23.1
20111001	23.3
20111001	23.7
20111001	23.9
20111001	23.5
20111001	23.3
20111002	23.1
20111002	23.0
20111002	22.9
20111002	23.0
20111002	23.5
20111002	23.7
20111002	23.4
20111002	23.1
20111003	23.0
20111003	22.9
20111003	22.9
20111003	23.2
20111003	24.2
20111003	24.4
20111003	23.6
20111003	23.3
20111004	23.1
20111004	23.0
20111004	23.0
20111004	23.3
20111004	23.8
20111004	23.9
20111004	23.5
20111004	23.4
20111005	23.2
20111005	23.0
20111005	23.1
20111005	23.4
20111005	23.8
20111005	23.8
20111005	23.5
20111005	23.4
20111006	23.2
20111006	23.1
20111006	23.0
20111006	23.3
20111006	23.8
20111006	23.7
20111006	23.4
20111006	23.1

20100910	24.6
20100910	24.6
20100910	24.5
20100910	24.5
20100910	24.5
20100910	24.6
20100910	24.5
20100910	24.5
20100911	24.5
20100911	24.4
20100911	24.4
20100911	24.3
20100911	24.4
20100911	24.5
20100911	24.6
20100911	24.5
20100912	24.5
20100912	24.3
20100912	24.2
20100912	24.2
20100912	24.3
20100912	24.5
20100912	24.5
20100912	24.6
20100913	24.5
20100913	24.5
20100913	24.3
20100913	24.4
20100913	24.4
20100913	24.6
20100913	24.6
20100913	24.7
20100914	24.6
20100914	24.5
20100914	24.5
20100914	24.4
20100914	24.5
20100914	24.6
20100914	25.0
20100914	24.8
20100915	24.8
20100915	24.7
20100915	24.6
20100915	24.6
20100915	24.5
20100915	24.8
20100915	24.9
20100915	24.9
20100916	24.6
20100916	24.5
20100916	24.5
20100916	24.5
20100916	24.5
20100916	24.5
20100916	24.5
20100916	24.7

20120206	14.3	20110122	14.9	20110509	17.0	20111007	22.9
20120206	14.2	20110122	14.8	20110509	16.8	20111007	22.9
20120207	14.1	20110122	14.9	20110510	16.6	20111007	22.9
20120207	14.1	20110122	14.8	20110510	16.5	20111007	23.2
20120207	14.0	20110122	14.8	20110510	16.5	20111007	23.5
20120207	14.1	20110122	14.8	20110510	16.6	20111007	23.4
20120207	14.3	20110123	14.8	20110510	17.3	20111007	23.3
20120207	14.4	20110123	14.8	20110510	17.4	20111007	23.0
20120207	14.5	20110123	14.8	20110510	17.0	20111008	22.8
20120207	14.4	20110123	14.8	20110510	16.8	20111008	23.0
20120208	14.4	20110123	14.8	20110511	16.5	20111008	23.0
20120208	14.5	20110123	15.0	20110511	16.2	20111008	23.0
20120208	14.4	20110123	15.0	20110511	16.3	20111008	22.9
20120208	14.4	20110123	14.8	20110511	16.8	20111008	22.9
20120208	14.6	20110124	14.8	20110511	17.1	20111008	22.6
20120208	14.6	20110124	14.7	20110511	17.1	20111008	22.4
20120208	14.5	20110124	14.8	20110511	17.0	20111009	22.4
20120208	14.5	20110124	14.6	20110511	16.9	20111009	22.3
20120209	14.4	20110124	14.7	20110512	16.9	20111009	22.3
20120209	14.4	20110124	14.7	20110512	16.8	20111009	22.3
20120209	14.4	20110124	14.8	20110512	16.6	20111009	22.2
20120209	14.4	20110124	14.7	20110512	16.7	20111009	22.2
20120209	14.6	20110125	14.6	20110512	16.9	20111009	21.9
20120209	14.6	20110125	14.5	20110512	17.0	20111009	21.3
20120209	14.5	20110125	14.6	20110512	16.8	20111010	21.4
20120209	14.3	20110125	14.7	20110512	16.8	20111010	21.3
20120210	14.3	20110125	14.8	20110513	16.9	20111010	21.2
20120210	14.3	20110125	14.8	20110513	16.9	20111010	21.2
20120210	14.3	20110125	14.7	20110513	17.0	20111010	21.3
20120210	14.3	20110125	14.7	20110513	17.4	20111010	21.2
20120210	14.3	20110126	14.4	20110513	18.1	20111010	20.9
20120210	14.3	20110126	14.3	20110513	18.6	20111010	20.7
20120210	14.3	20110126	14.5	20110513	18.0	20111011	20.4
20120210	14.3	20110126	14.6	20110513	17.6	20111011	20.3
20120211	14.3	20110126	14.7	20110514	17.4	20111011	20.5
20120211	14.4	20110126	14.8	20110514	17.3	20111011	20.8
20120211	14.3	20110126	14.8	20110514	17.2	20111011	20.9
20120211	14.3	20110126	14.7	20110514	17.7	20111011	21.0
20120211	14.3	20110127	14.5	20110514	18.9	20111011	20.9
20120211	14.4	20110127	14.4	20110514	19.1	20111011	20.8
20120211	14.4	20110127	14.4	20110514	18.3	20111012	20.8
20120211	14.3	20110127	14.5	20110514	18.3	20111012	20.8
20120212	14.2	20110127	14.5	20110515	18.0	20111012	20.8
20120212	14.3	20110127	14.6	20110515	17.9	20111012	21.0
20120212	14.4	20110127	14.6	20110515	18.1	20111012	21.2
20120212	14.3	20110127	14.5	20110515	18.4	20111012	21.2
20120212	14.5	20110128	14.3	20110515	19.0	20111012	21.0
20120212	14.4	20110128	14.3	20110515	19.1	20111012	20.8
20120212	14.4	20110128	14.3	20110515	18.5	20111013	20.7
20120212	14.4	20110128	14.4	20110515	18.2	20111013	20.7
20120213	14.2	20110128	14.6	20110516	17.6	20111013	20.6
20120213	14.0	20110128	14.8	20110516	17.5	20111013	20.7
20120213	14.1	20110128	14.7	20110516	16.6	20111013	21.1
20120213	14.3	20110128	14.6	20110516	15.7	20111013	21.3
20120213	14.3	20110129	14.6	20110516	15.9	20111013	20.9
20120213	14.4	20110129	14.6	20110516	16.0	20111013	20.8
20120213	14.3	20110129	14.6	20110516	15.9	20111014	20.8
20120213	14.3	20110129	14.7	20110516	15.8	20111014	20.7
20120214	14.1	20110129	14.7	20110517	16.7	20111014	20.6
20120214	14.1	20110129	14.6	20110517	16.9	20111014	20.7
20120214	14.1	20110129	14.7	20110517	16.9	20111014	21.0
20120214	14.1	20110129	14.7	20110517	17.0	20111014	20.9
20120214	14.0	20110130	14.6	20110517	17.4	20111014	20.5
20120214	14.0	20110130	14.5	20110517	17.7	20111014	20.2
20120214	13.9	20110130	14.4	20110517	17.1	20111015	20.0
20120214	13.9	20110130	14.5	20110517	16.9	20111015	20.1
20120215	13.8	20110130	14.8	20110518	16.8	20111015	20.0
20120215	13.8	20110130	14.8	20110518	16.7	20111015	19.6
20120215	13.8	20110130	14.8	20110518	16.8	20111015	19.5
20120215	14.0	20110130	14.7	20110518	17.2	20111015	19.5
20120215	14.2	20110131	14.7	20110518	17.8	20111015	19.5
20120215	14.1	20110131	14.6	20110518	17.8	20111015	19.4
20120215	14.0	20110131	14.5	20110518	17.5	20111016	19.5
20120215	14.0	20110131	14.5	20110518	17.3	20111016	19.5
20120216	14.0	20110131	14.8	20110519	17.2	20111016	19.4
20120216	13.9	20110131	14.9	20110519	17.0	20111016	19.4
20120216	14.0	20110131	14.8	20110519	17.0	20111016	19.8
20120216	14.0	20110131	14.7	20110519	17.5	20111016	19.9

20120216	14.1
20120216	14.0
20120216	13.9
20120216	13.9
20120217	13.9
20120217	13.8
20120217	13.7
20120217	13.7
20120217	14.0
20120217	14.0
20120217	13.8
20120217	13.8
20120218	13.9
20120218	13.9
20120218	13.8
20120218	13.9
20120218	14.4
20120218	14.6
20120218	14.3
20120218	14.1
20120219	14.0
20120219	13.9
20120219	13.8
20120219	13.8
20120219	14.3
20120219	14.5
20120219	14.2
20120219	14.0
20120220	14.0
20120220	14.0
20120220	13.9
20120220	13.9
20120220	14.3
20120220	14.4
20120220	14.3
20120220	14.2
20120221	14.1
20120221	14.1
20120221	14.0
20120221	14.0
20120221	14.1
20120221	14.1
20120221	14.1
20120221	13.9
20120222	13.9
20120222	13.8
20120222	13.7
20120222	13.6
20120222	13.7
20120222	13.7
20120222	13.7
20120222	13.8
20120223	13.8
20120223	13.8
20120223	13.8
20120223	13.9
20120223	14.2
20120223	14.2
20120223	14.1
20120223	14.0
20120224	13.9
20120224	13.9
20120224	13.9
20120224	13.8
20120224	14.3
20120224	14.6
20120224	14.3
20120224	14.2
20120225	14.1
20120225	14.1
20120225	14.1
20120225	14.1
20120225	14.5
20120225	14.6
20120225	14.3
20120225	14.2
20120226	14.0
20120226	14.0

20110201	14.6
20110201	14.6
20110201	14.4
20110201	14.4
20110201	14.5
20110201	14.4
20110201	14.2
20110201	14.1
20110202	14.2
20110202	14.3
20110202	14.2
20110202	14.1
20110202	14.4
20110202	14.6
20110202	14.5
20110202	14.2
20110203	14.1
20110203	14.2
20110203	14.1
20110203	14.2
20110203	14.6
20110203	14.6
20110203	14.5
20110203	14.4
20110204	14.4
20110204	14.4
20110204	14.3
20110204	14.3
20110204	14.4
20110204	14.5
20110204	14.5
20110204	14.3
20110205	14.3
20110205	14.3
20110205	14.2
20110205	14.3
20110205	14.7
20110205	14.8
20110205	14.7
20110205	14.5
20110206	14.4
20110206	14.4
20110206	14.4
20110206	14.4
20110206	14.7
20110206	14.8
20110206	14.8
20110206	14.6
20110207	14.5
20110207	14.4
20110207	14.5
20110207	14.6
20110207	14.8
20110207	14.9
20110207	14.8
20110207	14.7
20110208	14.6
20110208	14.6
20110208	14.5
20110208	14.5
20110208	15.0
20110208	15.0
20110208	14.8
20110208	14.7
20110209	14.6
20110209	14.6
20110209	14.6
20110209	14.6
20110209	14.8
20110209	14.8
20110209	14.8
20110209	14.7
20110210	14.6
20110210	14.6
20110210	14.6
20110210	14.6
20110210	14.6
20110210	14.7

20110519	18.3
20110519	18.5
20110519	18.0
20110519	17.8
20110520	17.7
20110520	17.4
20110520	17.4
20110520	18.1
20110520	18.7
20110520	19.0
20110520	18.2
20110520	18.2
20110521	18.1
20110521	18.0
20110521	17.9
20110521	18.3
20110521	18.7
20110521	18.6
20110521	18.4
20110521	18.3
20110522	18.1
20110522	18.1
20110522	18.2
20110522	18.2
20110522	18.2
20110522	18.3
20110522	18.2
20110522	18.1
20110523	18.0
20110523	17.9
20110523	17.7
20110523	18.1
20110523	18.6
20110523	18.6
20110523	18.3
20110523	18.1
20110524	18.1
20110524	18.1
20110524	18.1
20110524	18.2
20110524	18.6
20110524	18.7
20110524	18.6
20110524	18.3
20110525	18.2
20110525	18.2
20110525	18.2
20110525	18.4
20110525	19.2
20110525	19.1
20110525	18.9
20110525	18.6
20110526	18.5
20110526	18.5
20110526	18.5
20110526	18.7
20110526	19.2
20110526	19.6
20110526	19.1
20110526	18.9
20110527	18.8
20110527	18.8
20110527	19.0
20110527	19.3
20110527	20.6
20110527	20.4
20110527	19.8
20110527	19.3
20110528	19.1
20110528	19.8
20110528	19.7
20110528	20.0
20110528	20.8
20110528	21.8
20110528	21.0
20110528	20.3
20110529	20.1
20110529	19.3

20111016	19.5
20111016	19.3
20111017	18.8
20111017	18.9
20111017	18.8
20111017	19.0
20111017	19.3
20111017	19.4
20111017	19.1
20111017	18.9
20111018	18.6
20111018	18.7
20111018	18.8
20111018	18.8
20111018	19.2
20111018	19.2
20111018	19.0
20111018	18.8
20111019	18.6
20111019	18.6
20111019	18.5
20111019	18.8
20111019	19.2
20111019	19.4
20111019	19.1
20111019	19.0
20111020	18.7
20111020	18.5
20111020	18.4
20111020	18.7
20111020	19.1
20111020	19.1
20111020	18.8
20111020	18.6
20111021	18.6
20111021	18.6
20111021	19.1
20111021	19.4
20111021	19.1
20111021	19.0
20111021	18.7
20111021	18.6
20111022	18.6
20111022	18.5
20111022	18.6
20111022	18.9
20111022	19.1
20111022	19.1
20111022	19.0
20111022	18.9
20111023	18.8
20111023	19.1
20111023	19.6
20111023	19.9
20111023	20.3
20111023	20.5
20111023	20.3
20111023	20.1
20111024	19.7
20111024	19.4
20111024	19.6
20111024	19.7
20111024	20.1
20111024	20.1
20111024	19.9
20111024	19.7
20111025	19.5
20111025	19.5
20111025	19.6
20111025	19.9
20111025	20.3
20111025	20.3
20111025	20.2
20111025	20.1
20111026	20.0
20111026	19.9
20111026	19.8
20111026	19.9

20120226	13.9	20110210	14.6	20110529	19.1	20111026	20.0
20120226	14.0	20110210	14.5	20110529	19.5	20111026	20.0
20120226	14.4	20110211	14.4	20110529	20.2	20111026	20.0
20120226	14.6	20110211	14.4	20110529	20.3	20111026	19.9
20120226	14.4	20110211	14.4	20110529	20.1	20111027	19.9
20120226	14.2	20110211	14.5	20110529	19.8	20111027	20.0
20120227	14.1	20110211	14.6	20110530	19.6	20111027	20.0
20120227	13.9	20110211	14.6	20110530	19.6	20111027	20.2
20120227	14.0	20110211	14.6	20110530	20.1	20111027	20.4
20120227	14.0	20110211	14.5	20110530	20.2	20111027	20.4
20120227	14.0	20110212	14.4	20110530	21.2	20111027	20.2
20120227	13.9	20110212	14.4	20110530	21.2	20111027	20.1
20120227	13.7	20110212	14.6	20110530	20.7	20111028	20.1
20120227	13.7	20110212	14.5	20110530	20.4	20111028	20.0
20120228	13.7	20110212	14.6	20110531	20.2	20111028	19.9
20120228	13.7	20110212	14.5	20110531	20.2	20111028	20.1
20120228	13.7	20110212	14.3	20110531	20.5	20111028	20.3
20120228	13.8	20110212	14.3	20110531	21.0	20111028	20.4
20120228	14.1	20110213	14.3	20110531	21.8	20111028	20.1
20120228	14.2	20110213	14.2	20110531	22.9	20111028	19.9
20120228	13.9	20110213	14.2	20110531	22.1	20111029	19.8
20120228	13.7	20110213	14.2	20110531	20.8	20111029	19.8
20120229	13.7	20110213	14.4	20110601	20.8	20111029	19.5
20120229	13.7	20110213	14.6	20110601	20.6	20111029	19.8
20120229	13.7	20110213	14.4	20110601	20.8	20111029	20.2
20120229	13.7	20110213	14.2	20110601	21.3	20111029	20.2
20120229	14.1	20110214	14.1	20110601	21.9	20111029	20.0
20120229	14.3	20110214	14.0	20110601	21.9	20111029	19.9
20120229	14.1	20110214	14.0	20110601	21.3	20111030	19.7
20120229	14.1	20110214	14.2	20110601	21.0	20111030	19.7
20120301	14.1	20110214	14.6			20111030	19.6
20120301	14.0	20110214	14.8			20111030	19.7
20120301	14.0	20110214	14.6			20111030	19.9
20120301	14.1	20110214	14.4			20111030	19.9
20120301	14.6	20110215	14.2			20111030	19.7
20120301	14.7	20110215	14.2			20111030	19.6
20120301	14.4	20110215	14.2			20111031	19.5
20120301	14.2	20110215	14.4			20111031	19.7
20120302	14.1	20110215	14.6			20111031	19.6
20120302	14.0	20110215	14.8			20111031	20.0
20120302	14.0	20110215	14.6			20111031	20.2
20120302	14.1	20110215	14.4			20111031	20.2
20120302	14.4	20110216	14.3			20111031	20.0
20120302	14.4	20110216	14.3			20111031	19.8
20120302	14.2	20110216	14.3			20111101	19.8
20120302	14.2	20110216	14.4			20111101	19.8
20120303	14.1	20110216	14.5			20111101	19.8
20120303	14.0	20110216	14.5			20111101	19.8
20120303	14.0	20110216	14.5			20111101	20.2
20120303	14.1	20110216	14.5			20111101	20.2
20120305	14.7	20110217	14.5			20111101	20.1
20120305	14.8	20110217	14.5			20111101	19.8
20120305	14.4	20110217	14.4			20111102	19.6
20120305	14.3	20110217	14.5			20111102	19.7
20120306	14.2	20110217	14.7			20111102	19.6
20120306	14.2	20110217	14.7			20111102	19.8
20120306	14.2	20110217	14.6			20111102	20.0
20120306	14.2	20110217	14.6			20111102	20.1
20120306	14.3	20110218	14.6			20111102	19.9
20120306	14.4	20110218	14.6			20111102	19.8
20120306	14.2	20110218	14.5			20111103	19.7
20120306	14.1	20110218	14.5			20111103	19.7
20120307	14.1	20110218	14.6			20111103	19.7
20120307	14.1	20110218	14.6			20111103	19.8
20120307	14.0	20110218	14.6			20111103	20.1
20120307	14.0	20110218	14.6			20111103	20.1
20120307	14.1	20110219	14.6			20111103	19.9
20120307	14.2	20110219	14.6			20111103	19.8
20120307	14.1	20110219	14.6			20111104	19.6
20120307	13.9	20110219	14.6			20111104	19.5
20120308	13.8	20110219	14.7			20111104	19.5
20120308	13.9	20110219	14.7			20111104	19.5
20120308	13.8	20110219	14.7			20111104	19.9
20120308	13.8	20110219	14.6			20111104	19.9
20120308	14.0	20110220	14.6			20111104	19.6
20120308	14.3	20110220	14.7			20111104	19.5
20120308	14.2	20110220	14.6			20111105	19.5
20120308	14.2	20110220	14.7			20111105	19.5

20120309	14.1	20110220	14.7	20111105	19.6
20120309	14.2	20110220	14.8	20111105	19.6
20120309	14.2	20110220	14.8	20111105	19.8
20120309	14.3	20110220	14.7	20111105	19.8
20120309	14.6	20110221	14.6	20111105	19.6
20120309	14.6	20110221	14.6	20111105	19.4
20120309	14.5	20110221	14.6	20111106	19.3
20120309	14.3	20110221	14.6	20111106	19.4
20120310	14.2	20110221	14.7	20111106	19.3
20120310	14.2	20110221	14.7	20111106	19.4
20120310	14.1	20110221	14.6	20111106	19.7
20120310	14.2	20110221	14.5	20111106	19.6
20120310	14.4	20110222	14.5	20111106	19.6
20120310	14.6	20110222	14.5	20111106	19.5
20120310	14.3	20110222	14.6	20111107	19.5
20120310	14.2	20110222	14.6	20111107	19.3
20120311	14.1	20110222	14.7	20111107	19.2
20120311	14.0	20110222	14.8	20111107	19.4
20120311	14.0	20110222	14.8	20111107	19.6
20120311	14.2	20110222	14.6	20111107	19.5
20120311	14.4	20110223	14.4	20111107	19.5
20120311	14.4	20110223	14.2	20111107	19.5
20120311	14.3	20110223	14.2	20111108	19.5
20120311	14.2	20110223	14.2	20111108	19.4
20120312	14.0	20110223	14.2	20111108	19.4
20120312	13.9	20110223	14.2	20111108	19.5
20120312	13.9	20110223	14.2	20111108	19.5
20120312	13.9	20110223	14.2	20111108	19.5
20120312	14.2	20110224	14.1	20111108	19.4
20120312	14.4	20110224	14.1	20111108	19.3
20120312	14.1	20110224	14.2	20111109	19.3
20120312	14.0	20110224	14.2	20111109	19.2
20120313	13.8	20110224	14.3	20111109	19.2
20120313	13.8	20110224	14.3	20111109	19.3
20120313	13.8	20110224	14.3	20111109	19.3
20120313	14.0	20110224	14.3	20111109	19.3
20120313	14.4	20110225	14.2	20111109	19.3
20120313	14.8	20110225	14.1	20111109	19.3
20120313	14.5	20110225	14.2	20111110	19.2
20120313	14.2	20110225	14.3	20111110	19.2
20120314	14.1	20110225	14.3	20111110	19.1
20120314	14.0	20110225	14.3	20111110	19.2
20120314	13.9	20110225	14.2	20111110	19.5
20120314	14.1	20110225	14.1	20111110	19.5
20120314	14.8	20110226	14.0	20111110	19.3
20120314	14.9	20110226	14.0	20111110	19.2
20120314	14.6	20110226	13.9	20111111	19.1
20120314	14.3	20110226	14.0	20111111	19.1
20120315	14.2	20110226	14.3	20111111	19.1
20120315	14.1	20110226	14.2	20111111	19.1
20120315	14.0	20110226	14.1	20111111	19.4
20120315	14.2	20110226	14.2	20111111	19.6
20120315	14.8	20110227	14.1	20111111	19.4
20120315	15.1	20110227	13.9	20111111	19.3
20120315	14.6	20110227	13.9	20111112	19.2
20120315	14.4	20110227	14.0	20111112	19.1
20120316	14.2	20110227	14.5	20111112	19.0
20120316	14.1	20110227	14.5	20111112	19.2
20120316	14.0	20110227	14.3	20111112	19.6
20120316	14.2	20110227	14.2	20111112	19.6
20120316	14.6	20110228	14.1	20111112	19.2
20120316	15.3	20110228	13.9	20111112	19.1
20120316	14.7	20110228	13.9	20111113	19.1
20120316	14.4	20110228	14.0	20111113	19.1
20120317	14.3	20110228	14.4	20111113	19.0
20120317	14.0	20110228	14.4	20111113	19.1
20120317	13.8	20110228	14.2	20111113	19.3
20120317	14.1	20110228	14.2	20111113	19.2
20120317	14.9	20110301	14.1	20111113	19.0
20120317	15.7	20110301	14.0	20111113	18.8
20120317	14.9	20110301	14.0	20111114	18.6
20120317	14.7	20110301	14.0	20111114	18.6
20120318	14.6	20110301	14.1	20111114	18.5
20120318	14.5	20110301	14.2	20111114	18.5
20120318	14.4	20110301	14.2	20111114	18.9
20120318	14.6	20110301	14.1	20111114	18.8
20120318	15.3	20110302	14.2	20111114	18.6
20120318	15.7	20110302	14.1	20111114	18.6

20120318	15.6
20120318	15.0
20120319	14.8
20120319	14.7
20120319	14.7
20120319	14.8
20120319	15.3
20120319	15.9
20120319	15.8
20120319	15.4
20120320	15.2
20120320	15.0
20120320	14.8
20120320	15.0
20120320	15.6
20120320	16.3
20120320	15.8
20120320	15.4
20120321	15.3
20120321	15.0
20120321	14.9
20120321	15.1
20120321	15.5
20120321	15.9
20120321	15.4
20120321	15.1
20120322	15.0
20120322	14.9
20120322	14.8
20120322	15.0
20120322	15.5
20120322	15.9
20120322	15.2
20120322	14.9
20120323	14.7
20120323	14.7
20120323	14.6
20120323	14.8
20120323	15.9
20120323	16.3
20120323	15.6
20120323	15.1
20120324	14.8
20120324	14.8
20120324	15.0
20120324	15.3
20120324	16.4
20120324	17.0
20120324	15.9
20120324	16.0
20120325	15.8
20120325	15.3
20120325	15.1
20120325	15.5
20120325	15.9
20120325	15.6
20120325	14.8
20120325	14.6
20120326	14.7
20120326	14.7
20120326	14.8
20120326	15.3
20120326	16.4
20120326	16.3
20120326	15.7
20120326	15.4
20120327	15.1
20120327	14.9
20120327	14.9
20120327	15.2
20120327	16.1
20120327	16.7
20120327	15.8
20120327	15.3
20120328	15.1
20120328	15.1
20120328	15.1
20120328	15.5

20110302	14.1
20110302	14.2
20110302	14.5
20110302	14.6
20110302	14.5
20110302	14.3

20111115	18.5
20111115	18.5
20111115	18.6
20111115	18.8
20111115	19.1
20111115	19.0
20111115	18.7
20111115	18.7
20111116	18.6
20111116	18.5
20111116	18.5
20111116	18.5
20111116	18.7
20111116	18.5
20111116	18.3
20111116	18.2
20111117	18.3
20111117	18.3
20111117	18.2
20111117	18.3
20111117	18.5
20111117	18.5
20111117	18.3
20111117	18.1
20111118	18.1
20111118	18.1
20111118	18.1
20111118	18.2
20111118	18.5
20111118	18.4
20111118	18.2
20111118	18.2
20111119	18.3
20111119	18.3
20111119	18.3
20111119	18.4
20111119	18.6
20111119	18.5
20111119	18.3
20111119	18.1
20111120	18.1
20111120	18.0
20111120	18.2
20111120	18.4
20111120	18.5
20111120	18.6
20111120	18.4
20111120	18.4
20111121	18.2
20111121	18.1
20111121	18.1
20111121	18.3
20111121	18.4
20111121	18.4
20111121	18.2
20111121	18.1
20111122	18.1
20111122	18.1
20111122	18.1
20111122	18.0
20111122	18.0
20111122	17.9
20111122	17.8
20111122	17.8
20111123	18.0
20111123	17.9
20111123	17.9
20111123	18.1
20111123	18.3
20111123	18.2
20111123	18.1
20111123	18.1
20111124	18.1
20111124	18.1
20111124	18.1
20111124	18.2
20111124	18.6
20111124	18.7

20120328	16.2
20120328	16.5
20120328	15.7
20120328	14.9
20120329	14.9
20120329	15.1
20120329	15.3
20120329	15.6
20120329	16.6
20120329	16.8
20120329	16.1
20120329	15.4
20120330	15.3
20120330	15.3
20120330	15.5
20120330	15.8
20120330	16.5
20120331	16.7
20120331	16.4
20120331	15.9
20120331	15.7
20120401	15.5
20120401	15.4
20120401	15.3
20120401	15.6
20120401	16.1
20120401	17.0
20120401	16.7
20120401	16.2
20120402	16.0
20120402	15.9
20120402	15.9
20120402	16.1
20120402	16.6
20120402	16.9
20120402	16.5
20120402	16.2
20120403	16.1
20120403	15.9
20120403	15.7
20120403	15.9
20120403	16.5
20120403	16.7
20120403	16.5
20120403	16.3
20120404	16.3
20120404	16.2
20120404	16.1
20120404	15.8
20120404	16.2
20120404	16.7
20120404	16.4
20120404	16.1
20120405	16.0
20120405	15.7
20120405	15.7
20120405	15.9
20120405	16.4
20120405	16.8
20120405	16.4
20120405	16.1
20120406	15.8
20120406	15.9
20120406	15.9
20120406	16.0
20120406	16.5
20120406	16.9
20120406	16.4
20120406	15.9
20120407	15.9
20120407	15.8
20120407	15.8
20120407	15.9
20120407	15.9
20120407	15.9
20120407	15.7
20120407	15.3
20120408	15.1

20111124	18.7
20111124	18.5

20120408	15.2
20120408	15.3
20120408	15.5
20120408	15.6
20120408	14.9
20120408	14.8
20120408	15.3
20120409	15.3
20120409	15.1
20120409	14.7
20120409	14.7
20120409	15.0
20120409	15.0
20120409	15.0
20120409	15.0
20120410	15.0
20120410	15.0
20120410	15.0
20120410	15.2
20120410	15.8
20120410	15.9
20120410	15.9
20120410	15.5
20120411	15.3
20120411	15.1
20120411	15.1
20120411	15.4
20120411	15.8
20120411	16.0
20120411	15.9
20120411	15.7
20120412	15.5
20120412	15.5
20120412	15.5
20120412	15.8
20120412	15.9
20120412	15.9
20120412	15.7
20120412	15.8
20120413	15.7
20120413	15.6
20120413	15.5
20120413	15.4
20120413	15.7
20120413	15.9
20120413	15.8
20120413	15.8
20120414	15.8
20120414	15.5
20120414	15.4
20120414	15.5
20120414	15.5
20120414	14.8
20120414	14.6
20120414	15.2
20120415	15.2
20120415	15.2
20120415	15.3
20120415	15.4
20120415	15.7
20120415	15.9
20120415	15.7
20120415	15.5
20120416	15.5
20120416	15.3
20120416	15.3
20120416	15.4
20120416	15.5
20120416	15.6
20120416	15.4
20120416	15.3
20120417	15.3
20120417	15.2
20120417	15.1
20120417	15.4
20120417	15.9
20120417	15.9
20120417	15.6

20120417	15.3					
20120418	15.3					
20120418	15.2					
20120418	15.2					
20120418	15.3					
20120418	15.6					
20120418	15.5					
20120418	15.2					
20120418	15.3					
20120419	15.3					
20120419	15.3					
20120419	15.3					
20120419	15.4					
20120419	15.6					
20120419	15.5					
20120419	15.4					
20120419	15.4					
20120420	15.3					
20120420	15.4					
20120420	15.3					
20120420	15.4					
20120420	15.6					
20120420	15.5					
20120420	15.4					
20120420	15.4					
20120421	15.4					
20120421	15.4					
20120421	15.3					
20120421	15.4					
20120421	15.7					
20120421	15.9					
20120421	15.6					
20120421	15.5					
20120422	15.4					
20120422	15.4					
20120422	15.3					
20120422	15.7					
20120422	16.2					
20120422	16.5					
20120422	16.1					
20120422	15.9					
20120423	15.7					
20120423	15.7					
20120423	15.7					
20120423	15.9					
20120423	16.0					
20120423	15.9					
20120423	15.7					
20120423	15.5					
20120424	15.3					
20120424	15.1					
20120424	15.1					
20120424	15.6					
20120424	16.5					
20120424	16.8					
20120424	16.1					
20120424	15.7					
20120425	15.6					
20120425	15.6					
20120425	15.6					
20120425	15.9					
20120425	15.9					
20120425	15.9					
20120425	15.9					
20120425	15.9					
20120426	15.8					
20120426	15.8					
20120426	15.8					
20120426	16.1					
20120426	16.9					
20120426	17.1					
20120426	17.0					
20120426	16.9					
20120427	16.8					
20120427	16.6					
20120427	16.4					
20120427	16.9					
20120427	17.9					

20120427	18.1										
20120427	17.2										
20120427	16.2										
20120428	16.2										
20120428	16.3										
20120428	16.4										
20120428	16.9										
20120428	17.5										
20120428	18.0										
20120428	18.3										
20120428	17.8										
**AVG**	**15.6**	**AVG**	**15.7**	**AVG**	**16.2**	**AVG**	**22.8**	**AVG**	**23.5**	**AVG**	**25.6**
**SD**	**1.2**	**SD**	**1.3**	**SD**	**1.7**	**SD**	**3.0**	**SD**	**1.7**	**SD**	**1.1**
**n**	**1177**	**n**	**860**	**n**	**728**	**n**	**934**	**n**	**408**	**n**	**520**
**95%**	**0.1**	**95%**	**0.1**	**95%**	**0.1**	**95%**	**0.2**	**95%**	**0.2**	**95%**	**0.1**
**CI**	**15.5**	**CI**	**15.6**	**CI**	**16.0**	**CI**	**22.6**	**CI**	**23.4**	**CI**	**25.5**
**CI**	**15.6**	**CI**	**15.7**	**CI**	**16.3**	**CI**	**23.0**	**CI**	**23.7**	**CI**	**25.7**

Table S3. At-depth temperature data for the six experimental transplant periods. At-depth temperatures were obtained from linear regression analysis between depth temperature, recorded by temperature sensors placed in the sea bottom for almost 23 months (from June 3rd 2011 to April 25th 2013), and sea surface temperature, recorded by mareographic stations close to Panarea Island using SM3810 (Society for Environmental and Industrial monitoring; SIAP+MICROS) from the National Mareographic Network of the Institute for the Environmental Protection and Research (ISPRA, available to http://www.mareografico.it)
